# MEG data representing a gamma oscillatory response during the hold/release paradigm

**DOI:** 10.1016/j.dib.2019.103787

**Published:** 2019-02-26

**Authors:** Jonathan Levy, Jean-Francois Démonet

**Affiliations:** a IDC Herzliya, Herzliya, Israel; b Leenaards Memory Center, Department of Clinical Neurosciences, CHUV and University of Lausanne, Lausanne, Switzerland

**Keywords:** Hold/release paradigm, Magnetoencephalography, Gamma oscillations, Language, Working memory, Attention

## Abstract

The article presents magnetoencephalography (MEG) data from healthy participants while undergoing the Hold/Release paradigm. During the paradigm, participants visually perceived a sequence of two letter strings which either assembled into real words (Hold condition) or pseudowords (Release condition). If the first letter string was morphologically valid, they held their attention (and/or held the item in working-memory) to wait for the second string, whereas if it were invalid, they could release it, respectively. We present data on high-frequency neuronal oscillations of the Hold condition compared to the Release condition. Making this information publicly available could allow other researchers to perform analyses and contribute to understanding the cognitive processes such as language, mnemonic or attentional processes.

**Table:** Specifications table

Subject area	*Neuroscience*
More specific subject area	*Cognitive Neuroscience*
Type of data	*Tables and Figures*
How data was acquired	*Whole-head CTF MEG system with 275 DC SQUID axial gradiometers*
Data format	*Raw (Power) & Analyzed (statistical contrast of Neural oscillatory power)*
Experimental factors	*A group of young adults participated in the experiment while performing the Hold/Release paradigm.*
Experimental features	*Hold Release paradigm*
Data source location	*Nijmegen, The Netherlands*
Data accessibility	*Yes (*https://doi.org/10.17632/zpbxzpm6m6.1)
Related research article	J. Levy, P. Hagoort, J.F. Démonet, A neuronal gamma oscillatory signature during morphological unification in the left occipitotemporal junction, Hum. Brain Mapp. 35 (2014) 5847–5860. https://doi.org/10.1002/hbm.22589.

**Table:** 

**Value of the Data** • The present data offers a valuable contribution for researchers studying working-memory, language, attention and their underlying neural mechanisms. • The data represents a broad high-frequency response, which is often difficult to obtain non-invasively, in healthy subjects and can be used to perform several data analysis schemes. • The data can inspire other future or ongoing investigations by examining the present, data.

## 1 Data

The data presented in this manuscript is magnetoencephalography (MEG) data, which was partially used in a previous published experiment [1]; in that publication, three sub-conditions within the Hold condition were analyzed and reported. The data, in the currently presented article, were collected from healthy participants performing the Hold Release paradigm, which is thought to involve working memory, attention and language processes [1], [2]. We publish here (https://doi.org/10.17632/zpbxzpm6m6.1) the raw power data in the time-frequency spectrum (TFR) of −400 to 700 ms (a couple of subjects with fewer samples had −350 to 650 ms) post stimulus onset and at 40–150 Hz for the two conditions. Specifically, the data is organized in the following way: 30 matrices from 15 participants, with 2 matrices for each participant. Each matrix represents either the hold ('h') or the release ('r') condition. In each matrix you will find 4 dimensions: trials * MEG-sensors * spectral-frequency (40:5:150) * time (−0.4:.05:.7).

In addition, we statistically contrasted the two conditions (independent sample *t*-test). In Supplementary Table S1, data are organized as statistical TFR tables, containing t-values in each of the 23 lines (representing 40–150Hz) and in each of the 21 columns (representing −350 to 650 ms) for every one of the 15 subjects (all MEG sensors and trials averaged).

## 2 Experimental design, materials, and methods

**Fig. 1:**
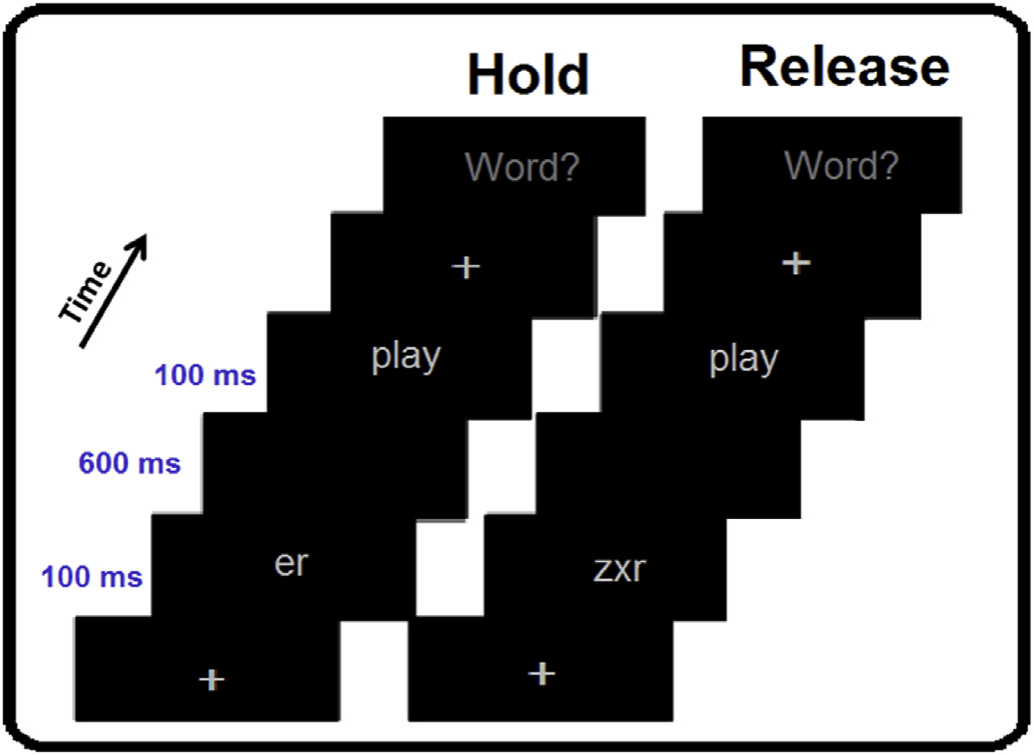
Experimental Design Experimental paradigm of the morphological unification procedure, prompted by a lexical decision task.

MEG data were collected from fifteen healthy right-handed and native-Dutch subjects while performing the Hold/Release paradigm (Fig. 1). As cab be illustrated in Fig. 1, pairs of stimuli were unified together, and then either represent real Dutch words, or not, respectively. Naturally, subjects are able to make their lexical decision earlier for Release trials compared to Hold trials. Hence, we retained data only from trials in which response was made after the onset of the second lexical item (i.e., stem). This ensured that data would not be contaminated or biased by motor response. To ensure equivalent comparison between trials, we matched the trial numbers between the two conditions, in a way that conditions with excessive trials were randomly discarded. On average, the number of trials in the hold condition was 29.93 and in the release condition 30, and there was no statistically significant difference (P = .94) between the trial numbers in the two conditions. The fully detailed Experimental information is described in our previous publication [1].

**Fig. 2:**
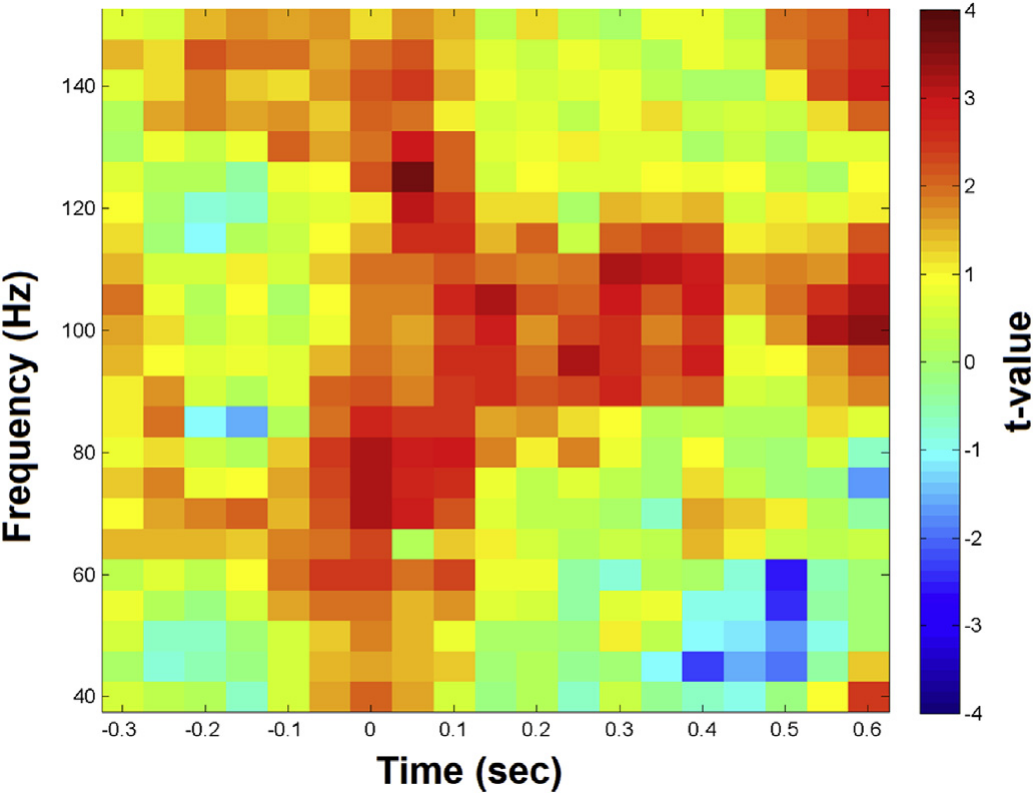
Hold vs Release Statistical contrast illustrated as a time-frequency representation.

We present the statistical contrast (independent sample *t*-test) TFR matrices of the two conditions (Hold vs Release) in Fig. 2, with all MEG sensors averaged. Data reveal a gamma frequency pattern: a broad-band pattern in the first 120 ms, and a narrow-band pattern at 120–420 ms (P _cluster-corrected_ < 0.05).

## References

[1] Levy J., Hagoort P., Démonet J.F. (2014). A neuronal gamma oscillatory signature during morphological unification in the left occipitotemporal junction. Hum. Brain Mapp..

[2] Martin C.D., Thierry G., Démonet J.F. (2010). ERP characterization of sustained attention effects in visual lexical categorization. PLoS One.

