# Supplementary material for: MEG data representing a gamma oscillatory response during the hold/release paradigm
Source: Data Brief. 2019 Feb 26;23:103787. doi: 10.1016/j.dib.2019.103787 (PMC6660567; doi:10.1016/j.dib.2019.103787)
Supplement: Supplementary file 1 — Multimedia component 1 [file mmc1.doc]

**Competing interests statement:**

The Authors declare no competing interests.
