# Supplementary material for: MEG data representing a gamma oscillatory response during the hold/release paradigm
Source: Data Brief. 2019 Feb 26;23:103787. doi: 10.1016/j.dib.2019.103787 (PMC6660567; doi:10.1016/j.dib.2019.103787)
Supplement: Multimedia component 1 [file mmc2.doc]

**Supplementary Data**

We present below 15 tables, one for each subject. In each table, columns represent time samples from -350 till 650 ms in jumps of 50ms, whereas rows represent frequency samples from 40 till 150 Hz in jumps of 5Hz.

| -1.9502 | 0.1761 | 2.437 | 0.7478 | -0.1392 | -0.655 | 1.6168 | 1.2456 | 2.7724 | -0.2258 | -0.3422 | -0.4141 | 0.1215 | -1.928 | 0.6846 | -1.6772 | 1.2214 | 2.5693 | 1.2215 | -0.5344 | -0.3185 |
| --- | --- | --- | --- | --- | --- | --- | --- | --- | --- | --- | --- | --- | --- | --- | --- | --- | --- | --- | --- | --- |
| 1.4201 | -0.0227 | 1.6961 | 1.7522 | 0.7745 | -0.0315 | 0.8296 | -0.6655 | 0.706 | -1.7371 | -0.0182 | -1.5738 | 0.0637 | 1.3638 | 1.496 | 1.2016 | 0.8834 | 0.088 | 1.9331 | 0.8727 | 0.4935 |
| 1.1686 | 1.5919 | 0.0103 | 1.2258 | 0.6082 | -1.2513 | -0.8386 | -0.8958 | 1.1783 | 0.2192 | -1.0409 | 0.8147 | -0.604 | 0.4648 | -1.1019 | 1.6044 | 2.3057 | -0.877 | 0.1999 | 1.2918 | -1.2357 |
| 0.249 | 1.0309 | 1.0051 | -1.2651 | -0.3133 | -0.2782 | 0.2747 | 0.5927 | -0.9121 | 2.1353 | -0.8588 | 0.3814 | 0.4609 | 0.4746 | -0.0521 | -0.2988 | 0.3275 | 0.3366 | -1.8973 | 0.5907 | -0.5764 |
| 0.3065 | -0.0753 | -1.2909 | -2.9607 | -0.2717 | 0.9874 | 0.3617 | 1.2581 | -0.417 | 0.6626 | 1.6762 | 0.0106 | 0.4417 | -0.2287 | -0.4054 | 0.3635 | -0.6033 | -0.2648 | -3.7635 | -0.2182 | 0.7481 |
| -1.5155 | -2.6265 | -0.5323 | -1.3398 | 1.5068 | 1.0482 | -2.189 | -0.2255 | 1.3582 | -1.2113 | 0.2938 | 0.1728 | 1.8478 | 0.9842 | -1.6492 | -1.2261 | -2.3598 | 0.0029 | -0.878 | 2.1951 | 0.2694 |
| -0.3056 | -0.2277 | 2.8672 | 1.1113 | 0.4172 | 0.4286 | 1.2568 | -0.0672 | 1.0638 | 1.407 | 2.76 | 0.8816 | -1.4812 | 0.0431 | -0.7636 | 1.0391 | -1.3702 | 1.4661 | 1.2204 | 0.736 | -0.4041 |
| 0.4111 | -1.2083 | 0.48 | 1.2589 | 1.1834 | -0.2663 | 1.5491 | 2.1363 | 1.8653 | 2.0066 | -0.297 | 0.3228 | -0.5477 | 1.3 | 0.5858 | 0.7906 | -1.5798 | 1.0532 | 1.2302 | 0.6477 | -1.5683 |
| -0.0105 | 2.0528 | 0.593 | 0.1575 | -0.3061 | 0.9079 | 0.5617 | 2.4617 | -0.192 | 0.1991 | -0.5494 | 0.1382 | -1.3917 | 0.6737 | 1.2415 | 0.6333 | 2.1565 | 1.0881 | -0.2169 | -0.8648 | 1.2091 |
| -0.3465 | 0.9003 | 2.1766 | 0.6712 | 0.0131 | 0.714 | -0.7761 | 0.2713 | -1.2085 | 0.7732 | -0.8707 | -0.6012 | 0.6219 | 1.5045 | 0.408 | 0.7143 | 0.728 | 2.6714 | -0.0543 | 0.2704 | 0.113 |
| 2.295 | -0.2955 | 1.5773 | 1.0971 | -1.4425 | -0.2631 | 0.1365 | 1.1306 | 0.2213 | -2.2417 | 0.8114 | 0.0804 | -0.1739 | -1.3272 | 0.4471 | 2.425 | -0.3906 | 0.8142 | 1.495 | -2.1596 | -0.2502 |
| 0.4169 | 0.9276 | -0.809 | -0.3826 | -0.2109 | 0.283 | 0.425 | -0.5612 | 1.6626 | -0.565 | 0.8493 | 0.1803 | 0.7419 | 0.6769 | -0.8172 | -0.339 | 0.2954 | -1.176 | 1.2367 | -0.5396 | 0.6304 |
| 0.6073 | -1.5369 | -2.538 | -0.2511 | 0.04 | 0.6676 | 1.0151 | -1.0156 | 0.459 | 0.5325 | 0.025 | 0.154 | 0.341 | -0.4352 | 1.4369 | 0.3301 | -0.455 | -2.9053 | 0.0774 | 0.7317 | 1.3516 |
| -2.5442 | -0.448 | -1.6925 | 1.3547 | 1.8466 | -0.8936 | -0.3264 | 1.5095 | -1.1396 | 0.3922 | 1.286 | 1.8531 | 1.3188 | -1.945 | -0.0342 | -2.7885 | -0.0862 | -1.5137 | 1.9294 | 0.6041 | 0.9595 |
| 0.1991 | 1.9619 | -0.4729 | 0.8039 | 0.1994 | 0.842 | -0.3801 | 0.6641 | 1.9991 | 2.2976 | 1.0955 | -0.9262 | 0.077 | -1.3663 | 0.7425 | -0.4014 | 1.1271 | -0.3333 | 0.7598 | -0.3145 | 2.0857 |
| -1.7764 | 0.0455 | 2.6491 | 1.0589 | -0.4792 | -0.1462 | 2.1201 | 1.6027 | 2.434 | 0.1122 | 0.5997 | -0.089 | 0.4291 | -0.877 | -0.5138 | -1.6321 | 1.1048 | 2.1964 | 0.3521 | -0.5129 | -0.9054 |
| 1.6735 | 0.3835 | 1.1771 | 0.8044 | 0.6246 | 0.2564 | 2.2226 | -0.0004 | 0.5308 | -1.1912 | -1.2267 | -1.4126 | 0.1998 | 1.2052 | 1.6794 | 1.8268 | 1.6996 | -0.0214 | -0.4999 | 1.5248 | 1.5898 |
| 0.0925 | 1.9039 | -0.4263 | -0.3985 | 0.8128 | -1.315 | -0.319 | -1.3207 | 0.9364 | -0.0292 | -0.6062 | 0.4258 | -0.7105 | 0.097 | -1.5252 | 0.2404 | 2.9034 | -1.0738 | 0.5751 | 0.1025 | -1.2256 |
| -0.3393 | 0.3744 | 0.9312 | -1.3537 | 0.4129 | -0.7102 | 1.4221 | 0.834 | -1.1725 | 0.4684 | -0.1532 | 0.4355 | -0.378 | 0.0361 | 0.5204 | -0.3262 | 0.4274 | 1.0234 | -1.7325 | 0.8416 | -1.0518 |
| 0.9482 | -1.3 | -0.9486 | -0.087 | -0.7571 | 0.7764 | -0.1514 | 0.7679 | -0.4847 | 0.2768 | 0.6109 | 0.6095 | 0.4032 | -0.1401 | -0.2633 | 0.1346 | -1.8259 | 0.0707 | -0.8246 | -0.5633 | 0.2331 |
| -1.2812 | -2.1391 | -0.6108 | -0.2956 | 0.9102 | 0.7729 | -1.4067 | 0.1729 | 1.0586 | -0.1369 | 0.3064 | 0.0619 | 0.402 | 1.2059 | -1.5105 | -1.6321 | -2.2278 | -0.1793 | 0.1431 | 1.5841 | 0.5014 |
| -0.1066 | -1.6871 | 2.9357 | 1.762 | 0.2852 | 0.2293 | 0.9544 | -1.164 | 1.1146 | 1.9166 | 2.6153 | 1.5073 | -1.8795 | 0.0296 | -0.8013 | 0.0507 | -1.5318 | 1.7148 | 0.6429 | 0.8028 | -0.8741 |
| 1.1807 | -0.648 | 0.5567 | -0.1981 | 1.5916 | 0.0633 | 1.3746 | 2.087 | 2.2162 | 1.3374 | -0.5007 | -0.3087 | -1.0007 | 1.1639 | 0.3724 | 0.9373 | -0.3014 | 0.946 | -0.0914 | 1.6616 | -0.3236 |

| 1.1922 | 1.4754 | 2.4455 | -0.3605 | -0.0866 | -0.3774 | 0.1796 | -0.6915 | -0.9535 | -1.2093 | 1.0044 | 0.9454 | 1.3408 | -0.9557 | 0.3391 | 1.333 | 1.0633 | 3.0339 | -0.7357 | 0.159 | -1.5042 |
| --- | --- | --- | --- | --- | --- | --- | --- | --- | --- | --- | --- | --- | --- | --- | --- | --- | --- | --- | --- | --- |
| 0.772 | -0.44 | 0.114 | -0.2926 | -0.1524 | -0.9338 | -0.5795 | 1.9591 | 2.534 | 1.6282 | 1.0655 | -0.2878 | 0.7808 | 1.1469 | 0.149 | 1.3549 | -1.3513 | 1.1414 | -1.066 | -0.7551 | -0.4113 |
| -0.8488 | -0.2367 | 0.9933 | 0.5446 | -0.5946 | 1.8031 | -1.9161 | -0.4064 | 0.5586 | 0.6698 | 1.5255 | 0.0138 | -0.1977 | 1.7103 | -2.3368 | 0.4262 | -0.0061 | 0.962 | 0.6837 | -0.0825 | 1.0594 |
| 0.8615 | 0.267 | 0.0407 | 2.0728 | -0.3244 | -0.9659 | 0.3262 | 0.2567 | 0.6034 | -0.3407 | 0.677 | -0.6162 | -1.8679 | 0.5393 | -0.1894 | 1.2792 | 0.7835 | 0.4754 | -0.7597 | -0.1789 | -1.2293 |
| 1.3398 | 0.6479 | -0.83 | 0.1698 | 2.117 | 0.9375 | -0.6475 | 0.4434 | 0.0347 | 0.2504 | -2.1276 | 0.6997 | -2.8367 | 1.8788 | 0.4727 | 2.3879 | -0.7731 | 0.5148 | -1.3669 | 0.7538 | 2.1968 |
| -0.0642 | -1.0722 | 0.6946 | 0.1787 | 0.453 | 0.5893 | 1.2566 | 1.6056 | -2.0633 | -0.9378 | -1.2152 | -0.1496 | -0.7468 | 1.591 | 1.1013 | 0.6643 | -1.2176 | -0.5884 | 1.2184 | 1.1115 | 0.3346 |
| 0.8369 | -0.6223 | 0.5368 | 0.7912 | 2.1095 | 1.0983 | -2.4709 | -0.2824 | 0.3017 | -0.1111 | -1.307 | 1.4226 | 0.6538 | 1.1228 | 1.1492 | 0.2474 | 1.1517 | -0.2879 | 0.672 | -0.3955 | 0.1252 |
| -0.2624 | 0.9664 | 1.6755 | 2.0297 | -1.745 | -0.7322 | 0.2219 | 0.3762 | 0.463 | 1.3959 | -2.0854 | 2.8366 | 1.0097 | 0.1032 | -0.9499 | -0.3203 | 0.8534 | 1.2393 | 1.795 | -1.2908 | -1.137 |
| 1.7322 | 2.0156 | -0.1486 | 0.1167 | -0.6515 | 0.1325 | 0.1272 | -0.7072 | 1.175 | 0.6177 | 0.696 | 0.8945 | -0.5588 | -0.4585 | 1.0376 | 2.8414 | 2.5961 | -0.8781 | 1.211 | -2.1528 | 0.0799 |
| -0.6365 | 0.0537 | -0.6403 | 0.2719 | -0.7817 | -0.235 | 1.4707 | 0.9984 | 0.8123 | 0.6687 | -0.1049 | 1.3598 | 0.3184 | 0.1472 | 1.0452 | -1.856 | 1.011 | -0.9822 | 0.7801 | -0.2341 | 0.4037 |
| -0.4648 | 1.3163 | 0.4746 | -1.3536 | 1.5226 | -0.3454 | -0.8103 | 0.0571 | -0.1058 | 1.6671 | -0.116 | 0.5332 | 1.2433 | -2.0629 | -1.2894 | -0.9726 | 1.2495 | 0.0874 | -0.8083 | 0.5626 | -0.7025 |
| 0.418 | 0.3192 | 1.5246 | -0.9408 | 0.1332 | 0.5124 | 1.3588 | 1.2789 | 0.0966 | -0.298 | -0.0143 | -1.9539 | 0.5283 | -0.5663 | 1.8686 | -0.3719 | 0.8261 | -0.5856 | -0.6475 | -1.026 | 0.2898 |
| 0.1799 | -0.9166 | 0.0732 | 1.0468 | 0.4139 | 1.0358 | 0.9103 | 0.0334 | 1.9137 | -0.918 | 0.0218 | -1.8844 | 1.1193 | -0.1393 | 2.3842 | -0.2168 | -0.3166 | -1.0237 | -0.457 | 2.4356 | 1.19 |
| -0.7238 | 0.8663 | 0.1041 | 0.4679 | 1.7479 | 1.7592 | 1.9 | -2.526 | -0.7304 | -1.5229 | -0.0994 | -1.9438 | 2.0073 | 1.5128 | 0.7487 | -0.5904 | -1.2425 | 1.3159 | 0.5052 | 1.2918 | 0.6138 |
| -0.7991 | 0.6108 | 0.8106 | 2.0131 | 0.0997 | -2.1605 | -1.4599 | -0.07 | 0.5348 | -0.4782 | 1.7392 | -0.8344 | 2.2943 | 0.5806 | 1.2278 | 0.769 | -0.0892 | 0.7746 | 0.1695 | -0.399 | -1.974 |
| 1.0335 | 1.5911 | 2.3363 | -0.7936 | 0.3562 | -0.373 | 0.157 | -0.3408 | -1.3717 | -2.6361 | 1.3407 | 1.606 | 1.1899 | -0.0831 | -1.6223 | 0.7064 | 0.9498 | 2.3673 | -0.9023 | -0.4049 | -1.1884 |
| 1.8625 | -0.3597 | 0.0228 | -0.5251 | -0.6886 | -0.299 | -1.0037 | 1.3405 | 1.8001 | 0.8081 | 0.8962 | -0.6447 | -0.5166 | 0.7306 | 0.8968 | 2.8937 | -1.1452 | 1.1106 | -0.0435 | 0.1391 | 0.6199 |
| -0.3122 | -0.1842 | 0.4284 | 0.2597 | -0.1753 | 1.8859 | -1.1587 | 0.456 | 0.4931 | -0.3197 | 1.1452 | -0.5043 | -0.4253 | 0.6929 | -1.8998 | 0.6534 | -0.4065 | 0.3394 | 0.651 | 0.6653 | 2.1226 |
| 1.2607 | 0.5574 | -0.5953 | 0.7405 | -0.2417 | -0.5737 | 1.3599 | -0.3703 | 0.8159 | -0.4553 | -0.0744 | 0.1589 | -2.0538 | -0.1271 | -1.3466 | 1.7468 | 0.3404 | -0.4165 | -0.4231 | -0.3035 | -0.9369 |
| 0.7087 | 0.8423 | -0.6319 | 0.517 | 0.6555 | 0.6446 | 0.3524 | -0.3425 | -0.8951 | -0.4696 | -2.5497 | 1.638 | -0.0138 | 0.8522 | 0.1623 | 1.4614 | -0.877 | -0.2535 | -0.7179 | -0.1912 | 1.495 |
| -1.484 | -0.195 | 0.6648 | 0.7626 | 1.4132 | 1.2616 | 0.5461 | 1.9894 | -1.6884 | -0.6523 | -1.3771 | 0.0413 | -1.3994 | 2.115 | 0.8519 | -0.0246 | -1.018 | -0.7374 | 2.0018 | 1.0765 | 1.2786 |
| 0.7951 | -0.5309 | 0.8291 | 1.6088 | 2.0406 | 1.8482 | -2.1057 | -0.611 | -0.5151 | -0.5491 | -2.0759 | 1.7618 | 1.3295 | 1.2197 | 0.2215 | -0.4107 | 0.8864 | 0.462 | 1.3554 | 0.31 | 0.4392 |
| 0.3196 | 0.4955 | 2.1953 | 0.6615 | -2.2604 | -1.1738 | -0.0756 | 1.4976 | 0.4518 | 1.4085 | -1.2855 | 2.9056 | 0.773 | 1.0572 | -0.4473 | -0.8442 | 0.817 | 1.047 | 0.4668 | -2.052 | -1.2646 |
|  |  |  |  |  |  |  |  |  |  |  |  |  |  |  |  |  |  |  |  |  |
|  |  |  |  |  |  |  |  |  |  |  |  |  |  |  |  |  |  |  |  |  |
| = |  |  |  |  |  |  |  |  |  |  |  |  |  |  |  |  |  |  |  |  |
|  |  |  |  |  |  |  |  |  |  |  |  |  |  |  |  |  |  |  |  |  |
| 0.1757 | -0.0694 | -2.1322 | -1.8332 | 0.9974 | 1.0006 | 1.3235 | 0.8141 | -0.4984 | 1.8325 | 1.158 | 3.9986 | -1.3023 | -1.3625 | -0.4491 | 1.1132 | -0.1048 | -1.6021 | -1.6121 | 0.7942 | 1.0891 |
| -0.4311 | 2.3694 | 1.7989 | 1.6346 | 2.8595 | -1.4585 | 1.9916 | 0.5632 | 1.9512 | 0.2503 | -1.508 | 0.5769 | 0.4874 | 0.4499 | 0.5281 | -0.1622 | 2.6376 | 2.0423 | 0.8775 | 3.3499 | -1.194 |
| -0.6801 | 0.8265 | 0.6262 | 0.1047 | 1.4963 | -0.8412 | -0.4369 | 1.086 | -2.5022 | -1.6156 | 0.5415 | 0.7906 | 1.0235 | 0.293 | 1.0226 | -0.7375 | -0.2034 | 0.4045 | 0.2467 | 1.2198 | -0.8605 |
| 0.5309 | -0.1489 | 0.5602 | 0.2721 | -0.1749 | -0.3238 | -1.6344 | 1.2206 | -1.2758 | 1.6933 | -0.1906 | -0.1229 | -0.8647 | -0.6289 | -2.0792 | -0.1992 | -0.0698 | 0.8304 | -0.7007 | -0.0064 | 0.2172 |
| 0.9884 | 0.2258 | 0.3775 | -0.3628 | -1.359 | 2.2862 | -2.7546 | 1.8351 | -0.9151 | 1.6899 | -1.378 | 0.4045 | -2.2543 | -0.4618 | 1.6601 | -0.0928 | 1.777 | 1.4582 | -1.2728 | -1.2914 | 0.1316 |
| 0.7848 | 1.9316 | -2.1585 | 0.4833 | -0.3455 | -1.2235 | -1.1219 | 1.4137 | 0.7657 | -0.3457 | -1.3534 | -1.3069 | 0.3213 | -0.1877 | 2.3813 | 0.9247 | 2.0175 | -1.5022 | -0.194 | -1.5335 | -1.4369 |
| -1.6838 | -0.6476 | 1.2229 | 0.0207 | -2.0975 | 2.0381 | 0.2805 | 0.7682 | 0.0257 | -1.6022 | 0.0422 | -1.2653 | 0.8366 | -0.8532 | 0.1797 | -1.3832 | -0.299 | 1.1232 | -0.791 | -1.5412 | 1.6437 |
| 0.0118 | 0.3043 | 0.6847 | 1.7968 | -1.8801 | 1.9908 | 0.0568 | -1.4496 | -0.0526 | -3.0712 | 0.9902 | 0.5919 | 2.2667 | -1.7795 | -1.9182 | 0.7554 | 0.2603 | 0.1476 | 1.7582 | -0.2638 | 1.797 |
| 0.0943 | -1.8209 | 1.3363 | 0.5276 | 1.0749 | 1.3873 | -0.1765 | -0.2723 | 1.8088 | 2.0892 | 3.3844 | -1.0427 | 0.9422 | -0.4019 | 0.8133 | 0.3696 | -1.0534 | 1.7342 | 0.2863 | 0.9544 | 3.0755 |
| 1.9841 | 0.115 | 1.2835 | 1.7429 | -1.0785 | 1.5223 | 0.4772 | 2.0749 | -0.0951 | -2.0233 | 0.4923 | -0.0056 | 1.0393 | -0.3306 | 0.8584 | 3.0549 | 0.9176 | 0.281 | 2.3238 | -0.8689 | 2.4166 |
| -0.0147 | 0.5386 | -1.5485 | 0.942 | -0.6321 | -0.8801 | 1.2973 | -2.1604 | -1.0765 | -0.6858 | 1.5273 | 0.3146 | 0.2665 | 0.3782 | 0.1718 | -0.0519 | 0.3012 | -0.995 | 0.8079 | -1.3777 | -0.2503 |
| 0.972 | 1.9394 | -0.0829 | -0.3458 | -0.4165 | -1.7735 | 0.7022 | -0.0775 | 1.6529 | -0.8137 | 0.8259 | -0.9464 | -0.2746 | -2.6878 | -0.8099 | 0.3792 | 1.6728 | 1.3215 | 0.2637 | -0.2324 | -1.285 |
| 0.3067 | -0.0937 | 2.034 | -1.4769 | 1.47 | -1.1082 | -0.1581 | -0.2804 | 2.47 | -0.868 | 0.0202 | -1.9649 | -0.1767 | 1.7607 | -0.3661 | 1.2782 | 1.0076 | 2.434 | -1.5618 | 0.3444 | -1.6175 |
| 2.4205 | -2.1356 | -0.8305 | -0.9936 | -1.4618 | -2.3537 | 2.2223 | 1.2403 | 0.2203 | -0.7961 | -0.9902 | 0.5875 | -0.5619 | 2.7507 | -0.1629 | 2.1574 | -1.4148 | -0.1103 | -1.4461 | -2.2095 | -2.1241 |
| -1.4073 | 0.9308 | 0.5033 | -1.6899 | 1.941 | 0.0202 | 1.2351 | -0.1027 | -0.8012 | -0.7093 | -1.4256 | 1.0328 | -0.4073 | -0.2632 | -1.9814 | -0.9582 | -0.0534 | 0.3598 | -1.4507 | 1.4644 | 0.5009 |
| 0.9042 | 0.1624 | -2.4747 | -3.643 | 1.0976 | 0.6514 | 0.326 | 0.872 | -1.4422 | 0.9826 | 0.7674 | 3.2874 | -0.7771 | -2.9325 | 0.9207 | 0.6377 | -0.5998 | -1.986 | -0.8712 | 1.0355 | 0.9016 |
| -0.8117 | 1.9881 | 1.3101 | 1.2853 | 1.9456 | -1.3828 | 0.966 | 0.8675 | 2.2051 | 3.1799 | -0.8977 | 0.4116 | 1.1184 | 0.0923 | -0.0504 | -0.5655 | 3.0595 | 1.8715 | 0.762 | 4.2044 | -0.8548 |
| -0.55 | 0.9624 | 0.1086 | -1.1972 | 1.3459 | -0.661 | 1.0979 | 0.8098 | -2.0126 | 0.5457 | 0.36 | 1.1454 | 0.9062 | 1.2809 | 1.2602 | -0.1005 | -0.4387 | 0.441 | -0.8074 | 1.3905 | -0.6884 |
| 1.0438 | -1.4772 | 0.5931 | -0.8425 | -0.6716 | -0.0643 | -1.7759 | -0.5093 | -1.2357 | 2.3445 | 0.35 | -0.2953 | -1.0965 | -0.2511 | -1.6682 | 0.9061 | -1.1841 | 0.7217 | -0.865 | -0.0317 | 0.3528 |
| 1.8177 | -0.4926 | 0.3866 | -0.4054 | -1.7162 | 1.8478 | -0.2804 | 1.4906 | -0.8945 | 1.1907 | -1.0609 | -0.1779 | -2.3518 | -0.2847 | 0.9174 | 0.7935 | 1.0042 | 0.748 | -0.1974 | -1.2428 | 0.7444 |
| 1.056 | 2.4145 | -1.4465 | 0.2898 | -1.0349 | -0.7015 | -0.7761 | 2.2501 | -0.485 | -0.1456 | -1.9171 | -1.5571 | 1.6905 | -0.0526 | 1.5103 | 0.7173 | 2.1013 | -1.3383 | -0.1852 | -1.3215 | -1.2475 |
| -2.0061 | -0.5405 | 0.4879 | -1.3427 | -2.4933 | 2.1121 | 1.585 | 0.7311 | 0.1447 | -1.0885 | 0.1241 | -0.8879 | 1.6148 | -1.0339 | 1.0277 | -1.4428 | 0.1206 | 0.4634 | -1.8087 | -2.0083 | 2.6208 |
| 0.571 | 0.9983 | 0.6449 | 2.0686 | -1.0472 | 2.1904 | -0.0798 | -1.2969 | -1.1194 | -2.3514 | 0.9027 | -0.2507 | 1.2019 | -1.8749 | -1.8527 | 0.2902 | 1.055 | -0.3758 | 2.1486 | 0.4434 | 2.5816 |
|  |  |  |  |  |  |  |  |  |  |  |  |  |  |  |  |  |  |  |  |  |
|  |  |  |  |  |  |  |  |  |  |  |  |  |  |  |  |  |  |  |  |  |
| = |  |  |  |  |  |  |  |  |  |  |  |  |  |  |  |  |  |  |  |  |
|  |  |  |  |  |  |  |  |  |  |  |  |  |  |  |  |  |  |  |  |  |
| 2.1153 | 0.1689 | -0.102 | 1.1157 | -0.4157 | 3.3696 | -0.0994 | -1.4686 | -1.4282 | 0.2558 | -0.017 | -0.7187 | -2.551 | 1.2697 | 2.0912 | 2.9384 | 0.5143 | -0.0288 | 0.1467 | 0.687 | 2.1204 |
| 2.3277 | -0.6659 | 0.7751 | 1.4608 | -1.0682 | 0.2892 | -0.1543 | -0.1754 | 1.7562 | 1.1421 | 2.533 | 0.3547 | 0.8 | 2.4179 | 0.0619 | 1.2496 | -0.0367 | 1.1513 | 1.3211 | -1.1274 | -0.6055 |
| -0.4493 | 1.4567 | -3.2062 | -2.7964 | -0.3631 | 1.7223 | 0.0553 | 0.3285 | 1.166 | -0.3357 | -1.4951 | -0.5448 | 1.179 | 0.6511 | 0.927 | -0.6527 | 1.2599 | -1.883 | -2.9635 | -0.8931 | 1.4152 |
| -1.5978 | 1.7113 | -0.0346 | 3.0282 | -0.0696 | 0.1481 | -0.1054 | 0.3919 | -4.8389 | -0.5967 | 0.0395 | 0.9022 | 0.8189 | 0.2968 | 0.0438 | 0.9119 | 0.2045 | -0.6829 | 1.1659 | 0.1781 | -1.6582 |
| -2.228 | 1.7104 | -0.6765 | 0.2881 | -1.2212 | -0.6878 | -3.3109 | 0.9688 | 0.9824 | -0.343 | 1.6894 | 0.1904 | -0.5188 | -0.4578 | -0.1066 | -2.633 | 0.6313 | -0.6989 | -0.6291 | 0.1414 | -0.4624 |
| -1.6333 | 1.2138 | 0.7565 | -0.6833 | -1.6136 | -0.8796 | 1.1712 | -0.2059 | 1.9147 | 0.2323 | 2.4258 | -0.8737 | 0.8133 | -3.1632 | -1.5345 | -1.7006 | 1.6568 | 1.9122 | 1.4513 | -1.5401 | 0.3449 |
| -0.1003 | 0.9432 | -0.238 | -1.6409 | 0.8514 | -0.4413 | 0.7061 | -0.2017 | 1.1279 | -0.6018 | 0.4943 | 1.0269 | -0.4321 | 0.2091 | 2.3358 | -0.4797 | 0.3717 | 0.3617 | -0.1277 | 0.9069 | 0.1714 |
| 0.3902 | -0.4431 | 0.3873 | -2.3271 | 0.838 | -0.3143 | 2.5942 | -0.8068 | -1.5805 | 1.1107 | -0.0755 | -0.0026 | 1.9367 | -0.7196 | 1.771 | 0.5651 | 0.9161 | 2.2251 | -1.3273 | 0.7114 | 0.3268 |
| -0.6928 | 1.1036 | 1.731 | 1.2551 | 3.2501 | -0.6308 | 0.1132 | 0.4674 | 0.1974 | 0.8328 | -0.0416 | 1.7365 | 1.1228 | 1.5825 | 3.5093 | -0.1424 | 0.6218 | 0.8552 | 0.6523 | 2.0484 | -0.0066 |
| -1.0993 | 1.7061 | 1.0361 | -2.2317 | 0.3831 | -0.4524 | 1.4336 | 0.4289 | 1.2511 | 2.4734 | 0.4372 | 0.087 | 1.3869 | 0.1031 | 1.1864 | 0.6607 | 1.3219 | 1.2938 | -1.8021 | -0.5238 | -1.1533 |
| 1.8881 | -2.5673 | -1.4174 | -1.1277 | 2.4968 | 0.2806 | 0.3703 | 0.5821 | 0.2687 | -1.2705 | -1.0448 | -0.7729 | 0.1172 | 0.2293 | -0.9485 | 1.4474 | -0.9201 | -1.2369 | -0.68 | 1.9056 | 0.0493 |
| -0.0407 | 1.2596 | 2.0713 | -1.6385 | -0.0593 | 0.4621 | 0.4705 | -4.5912 | -0.5752 | 1.1082 | 1.3443 | 1.219 | 0.0799 | -0.8847 | 0.4024 | -1.0101 | 0.1298 | 1.4442 | 0.0575 | -1.6606 | 0.2638 |
| 0.4652 | -1.32 | 1.2793 | -0.5407 | -0.4259 | -2.4518 | 1.1715 | 1.9494 | -0.7935 | 1.0791 | -0.1314 | 2.5534 | 0.6308 | 0.2901 | -2.4529 | 0.8445 | -1.4304 | 0.237 | -0.0857 | 0.355 | -2.9483 |
| 2.074 | 0.5501 | 0.2853 | -0.846 | -0.5186 | 1.2198 | -0.2501 | 2.0837 | -0.5014 | 2.0889 | -0.9276 | 0.7654 | -3.0968 | -1.5151 | -1.0163 | 1.7839 | 1.107 | 0.9155 | -0.9881 | 0.8574 | 0.254 |
| 1.2051 | 0.1813 | -0.5303 | 0.0301 | -0.1492 | 0.9266 | -0.3223 | 0.4939 | -0.8036 | 0.1617 | 0.5902 | 0.1184 | -0.1134 | 1.9444 | -0.0893 | 0.8039 | 0.8946 | -0.0733 | 1.0147 | 0.0937 | 1.1577 |
| 0.2976 | 0.6381 | -1.4223 | 0.451 | -0.5963 | 2.8351 | -0.3413 | -2.2389 | -0.2705 | 0.0479 | -0.5856 | -1.5681 | -1.0359 | 0.9637 | 1.3007 | 1.3057 | 1.2108 | -0.53 | 0.3998 | 0.3738 | 2.3861 |
| 1.1508 | 1.511 | 0.8997 | 2.5592 | -0.2571 | 0.449 | -0.2658 | -0.3141 | 0.7328 | 1.2044 | 2.3258 | 0.9229 | 1.0951 | 3.3997 | -0.226 | 1.8743 | 0.9625 | 0.9391 | 1.358 | -0.1088 | -0.9277 |
| 1.2985 | 1.2786 | -2.6653 | 0.7528 | -0.0243 | 1.6097 | 0.5693 | 1.5939 | 1.8886 | -0.5849 | -0.5839 | -0.1537 | 0.3783 | 0.3644 | 2.183 | 1.1174 | 1.2665 | -1.5664 | 0.0105 | -0.9054 | 1.5746 |
| -1.8006 | -0.7779 | -0.2781 | 3.1142 | 0.2197 | -0.7265 | -1.1533 | -0.1686 | -3.5881 | -0.8063 | 0.1816 | 1.2452 | 0.3467 | -0.5002 | 0.3792 | -0.5522 | -0.7825 | -1.1377 | 2.1318 | 0.0118 | -1.4238 |
| 1.3236 | 1.8276 | -1.3939 | -0.0175 | -0.2347 | -0.1851 | -3.384 | -0.2128 | 1.046 | 0.2789 | 1.1944 | 0.3501 | -0.0402 | -0.498 | -0.6644 | 0.0075 | 1.1238 | -0.7964 | -1.23 | 0.5025 | 0.2323 |
| -1.426 | 1.4955 | 0.2258 | -0.7148 | -1.7926 | 0.3518 | 1.6889 | -0.3501 | 1.242 | 0.6789 | 3.1115 | -1.0137 | -0.0489 | -3.0713 | -0.7342 | -1.2142 | 1.4893 | 0.9096 | 0.5298 | -2.3386 | 1.1069 |
| 1.4003 | 0.6196 | -0.7958 | -0.4345 | 1.2218 | -0.1532 | 1.921 | -0.653 | 1.7442 | -0.5838 | 0.8071 | 1.0308 | -1.5963 | 0.337 | 2.7678 | 0.1415 | 1.2095 | 0.5915 | 0.9996 | 1.2096 | -0.3341 |
| 0.238 | -0.4894 | -0.1039 | -1.4936 | 1.2013 | -0.6622 | 1.8629 | -1.3162 | -0.9655 | 0.191 | -0.0143 | 0.0225 | 1.6729 | -0.3874 | 2.4998 | 0.266 | -0.1338 | 2.0792 | -0.514 | 1.307 | -0.2781 |
|  |  |  |  |  |  |  |  |  |  |  |  |  |  |  |  |  |  |  |  |  |
|  |  |  |  |  |  |  |  |  |  |  |  |  |  |  |  |  |  |  |  |  |
| = |  |  |  |  |  |  |  |  |  |  |  |  |  |  |  |  |  |  |  |  |
|  |  |  |  |  |  |  |  |  |  |  |  |  |  |  |  |  |  |  |  |  |
| 0.1842 | -2.2477 | -1.1226 | 0.9844 | -0.8773 | -0.021 | -1.7702 | 1.7461 | 3.0099 | 3.7569 | 1.5726 | 1.1454 | 0.11 | 0.6721 | 2.3991 | -0.3548 | -0.9176 | -0.9361 | 0.7538 | -0.2731 | 0.5099 |
| -0.7493 | 0.92 | -0.4375 | 1.6972 | 1.9079 | 1.7067 | 1.047 | 3.1253 | 0.1422 | 0.2995 | 0.3798 | 1.5959 | 1.0208 | -0.8486 | 0.5845 | -1.3204 | 1.2257 | 0.3036 | 0.6466 | 2.1128 | 2.2462 |
| -0.3421 | 0.2126 | 1.8767 | 1.0604 | -2.1867 | -1.3308 | 2.5565 | 0.568 | 0.967 | -1.0434 | 1.6368 | -0.992 | 1.2524 | -0.2465 | 1.7769 | 0.744 | 0.8704 | 1.3109 | 0.3569 | -1.505 | -0.9241 |
| 0.1397 | 0.6552 | -3.9742 | 0.202 | -0.491 | 2.1 | 0.8246 | -0.3428 | 0.0762 | 1.3023 | 0.1351 | -0.0814 | -0.4596 | 0.2833 | -2.298 | 0.6538 | 0.4914 | -1.9432 | 0.862 | 0.9155 | 1.7811 |
| -5.1464 | 2.0515 | 0.0642 | -0.1242 | 2.4002 | 0.4699 | -0.2839 | 0.9904 | -1.4991 | -2.8455 | 0.0091 | -0.4872 | -0.774 | 0.1118 | 1.3475 | -2.4743 | 2.5596 | 0.7484 | -0.5133 | 0.3773 | 0.6066 |
| -0.5236 | -0.9973 | 2.2956 | 0.6116 | 2.0631 | -0.9382 | 0.1046 | -3.0077 | 0.1281 | -1.9832 | 0.9008 | 0.9469 | 0.9851 | -0.4987 | 1.7574 | -0.1996 | -1.3035 | 1.3043 | 2.694 | 1.8832 | -1.5512 |
| 1.0424 | 0.4185 | 1.9077 | -0.8064 | 0.7192 | 0.171 | -0.5056 | -0.1835 | 1.6346 | 0.6566 | 0.7326 | 0.3237 | 1.4368 | 0.0819 | -0.3516 | -0.1825 | 0.7287 | 2.2112 | -1.8011 | 0.661 | -0.5301 |
| 2.4636 | 0.5171 | -0.6774 | 0.4389 | 0.7429 | -0.982 | 1.4206 | 1.2643 | 2.1301 | 1.4331 | 2.6433 | 2.4712 | 1.0824 | 0.358 | 0.1598 | 2.6551 | -1.5921 | -0.5163 | -0.2138 | 0.463 | -0.9467 |
| -0.2691 | 0.1645 | -0.2501 | -0.647 | 0.1775 | 2.0152 | 2.0229 | 2.3066 | 3.8442 | 0.2132 | 0.6935 | 0.4545 | 0.8882 | 1.9333 | -0.2847 | 0.6388 | -0.3734 | -0.363 | -0.3804 | 0.4971 | 1.8784 |
| 2.5796 | 0.2404 | 1.7184 | 2.14 | 1.7733 | -1.3217 | 2.049 | 0.7952 | 0.0607 | 1.0981 | 0.9773 | 0.6782 | -1.4322 | 0.4719 | -0.9594 | 1.1366 | 0.39 | 0.5063 | 2.2792 | 0.6529 | -0.8711 |
| -0.9174 | 1.1194 | 1.4499 | -2.0762 | -1.6284 | -1.0968 | 0.4988 | 0.6659 | -1.0796 | 1.85 | -1.0297 | 0.5073 | -0.7344 | 1.5907 | 0.2392 | -1.0994 | 0.6316 | 0.4896 | -1.8393 | 0.1545 | -0.1496 |
| 0.5325 | -4.4515 | 1.2097 | 0.1464 | 1.8909 | 1.4827 | 0.0439 | 0.1131 | 1.0712 | -0.3661 | -0.4913 | -0.1498 | 0.0397 | -1.9455 | 0.4932 | 1.0686 | -2.2614 | 1.5597 | 1.1795 | 1.3162 | 0.7071 |
| 1.3913 | 1.377 | -0.3324 | 2.5373 | 0.3125 | 2.2232 | 1.6149 | -1.4287 | -3.41 | 0.6423 | -0.583 | -0.5611 | 0.3819 | 0.8362 | -2.0794 | 2.3442 | 1.3577 | -1.1071 | 0.9721 | 1.8541 | 2.1137 |
| 0.4533 | 1.6803 | 0.8172 | 1.9972 | -1.2812 | 1.1865 | -3.3937 | -0.5619 | -1.9436 | 1.324 | 0.7767 | 3.4565 | -0.3963 | 1.6067 | 0.3328 | 0.0035 | 1.0946 | 2.6743 | 2.4992 | -1.6948 | 1.1011 |
| 0.7519 | 1.2791 | -0.6649 | 0.3559 | -0.5768 | 0.5413 | -0.336 | 0.9828 | 1.9203 | 0.5174 | 0.9625 | 1.5724 | 1.3065 | -0.0066 | -0.0287 | 0.8361 | 1.9773 | -2.1771 | -0.5444 | -0.5378 | -0.1236 |
| 0.4225 | -1.7137 | -0.9401 | 1.0952 | -1.3296 | -0.6745 | -0.3109 | 1.5069 | 3.6831 | 2.4118 | 1.2325 | 0.9089 | 0.4377 | 0.6823 | 2.6794 | -0.6629 | -1.6888 | -1.1247 | 0.2625 | -0.6467 | -0.0539 |
| -0.7617 | 0.2646 | -1.1324 | 1.1615 | 2.0026 | 0.9649 | 1.4627 | 3.5843 | -0.142 | 0.2243 | 0.788 | 1.516 | 0.8402 | -0.26 | 0.6578 | -0.5846 | -0.5526 | 0.2083 | 0.1596 | 1.6487 | 1.1616 |
| -0.1815 | 1.432 | 2.1992 | 0.0681 | -1.5807 | -0.7902 | 1.5287 | -0.3986 | 1.6581 | 0.2755 | 0.6755 | -1.0643 | 0.8368 | 0.081 | 1.0729 | 1.0987 | 0.6854 | 1.9217 | 0.2348 | -1.0548 | 0.039 |
| 0.0946 | 0.9652 | -2.6866 | -0.3174 | -0.8729 | 1.3725 | 0.6839 | -1.0289 | 0.6958 | -0.2404 | 0.8384 | -1.0547 | 1.9203 | 0.545 | -1.6084 | 0.1875 | 1.6029 | -2.1482 | 0.6013 | 0.0985 | 1.6442 |
| -4.5137 | 2.0487 | 0.4612 | 0.4084 | 1.8352 | 1.0334 | 0.2857 | 1.2717 | -1.1974 | -0.7653 | -0.4233 | -0.5943 | -1.7681 | 0.1854 | 1.3102 | -1.869 | 2.2691 | 0.7276 | -0.1666 | 0.3991 | 0.5484 |
| -0.6563 | -0.4022 | 2.5302 | 0.6332 | 2.1014 | -0.9392 | -0.8715 | -3.6671 | -0.3498 | -1.7579 | -0.1296 | 0.792 | 1.651 | -1.9707 | 2.3707 | 0.6044 | -1.0527 | 1.3606 | 1.7131 | 2.2184 | -1.3359 |
| 2.997 | 0.6841 | 2.3198 | -0.6877 | 1.6681 | 0.7297 | -0.9539 | -0.3249 | 1.1257 | 0.4193 | 1.3183 | 0.3697 | 2.5347 | 0.5597 | -0.8556 | 0.526 | 1.4976 | 2.9624 | -1.7989 | 1.1356 | 0.2414 |
| 1.8283 | -0.4432 | -0.3199 | -0.2154 | 1.2381 | -0.6629 | 0.6483 | 2.3174 | 2.4578 | 0.6518 | 1.6556 | 2.9613 | 0.1642 | 0.2309 | 0.2423 | 2.183 | -2.0326 | -1.033 | -0.0156 | 0.5184 | -0.2324 |
|  |  |  |  |  |  |  |  |  |  |  |  |  |  |  |  |  |  |  |  |  |
|  |  |  |  |  |  |  |  |  |  |  |  |  |  |  |  |  |  |  |  |  |
| = |  |  |  |  |  |  |  |  |  |  |  |  |  |  |  |  |  |  |  |  |
|  |  |  |  |  |  |  |  |  |  |  |  |  |  |  |  |  |  |  |  |  |
| -0.3376 | 2.2066 | 2.9796 | 3.18 | 0.6391 | 0.4506 | 0.6017 | 0.1253 | 2.0186 | -1.3591 | 0.6527 | -1.4955 | -0.0007 | 0.3322 | 0.3481 | 1.0648 | 2.0419 | 1.195 | 2.5839 | 0.9836 | 0.0549 |
| 0.9725 | 2.5523 | -0.1311 | 1.0348 | -0.0743 | 0.5508 | 1.0146 | -1.2314 | 1.0989 | 0.0849 | 1.0301 | -0.1829 | 0.4632 | 1.6564 | 1.9746 | 0.6226 | 2.5037 | -0.0511 | 0.7834 | 1.2704 | -0.4139 |
| 1.2125 | 0.7269 | 0.488 | -0.9114 | 2.6459 | -0.9853 | 0.9386 | 0.7059 | 1.857 | 0.9194 | -0.2709 | 0.8901 | -0.3571 | -0.2358 | 0.9007 | 0.8175 | 0.3576 | 0.2788 | -0.6488 | 2.2171 | -0.7851 |
| 1.2591 | -0.5092 | 0.818 | 1.5317 | 0.9068 | 0.9857 | 0.165 | 0.9272 | -2.1566 | 0.8665 | 0.4753 | 0.2552 | 1.781 | 0.8412 | 1.1558 | 0.126 | -0.5568 | -0.0839 | 0.8042 | 0.4546 | 0.7976 |
| -0.4773 | 0.4435 | -2.0804 | -2.4377 | 0.9338 | -1.2135 | -1.2241 | 0.1947 | 2.6204 | -0.5908 | 3.4006 | 1.0327 | -0.6653 | -1.464 | 0.0009 | -1.345 | -0.0587 | -0.6842 | -1.1011 | 0.4549 | -0.7721 |
| -0.8556 | -2.2965 | -0.8886 | -1.2919 | 0.8765 | 0.8037 | 3.3467 | -0.6797 | 2.8045 | -0.83 | -1.7536 | -0.373 | 2.4183 | 3.5428 | -0.9602 | -0.0463 | -1.0599 | -0.3391 | -1.6168 | 0.961 | 2.1903 |
| -0.692 | 0.4801 | 0.8223 | 1.4711 | 0.8298 | 1.5296 | 2.2334 | -0.6334 | 0.2108 | -0.1935 | 1.4261 | 3.5374 | -1.8032 | 0.3881 | 0.3484 | -0.4269 | 0.1501 | 0.3151 | 1.9919 | 1.4538 | 1.1967 |
| 1.4421 | 1.7229 | 2.225 | 1.1882 | 2.6468 | 1.8945 | 1.2064 | 0.514 | 0.3903 | 2.6199 | -2.0813 | 0.5921 | -1.2472 | -0.0586 | 0.4701 | 1.3079 | 1.742 | 2.1503 | 0.0304 | 1.554 | 1.1455 |
| 2.9069 | 2.2623 | 3.5858 | 0.0392 | 0.3021 | 1.5206 | 0.2441 | 1.9247 | -1.2261 | 0.8011 | -1.5793 | 0.1992 | 0.2349 | 0.3403 | 2.0328 | 1.7649 | 0.8989 | 2.2205 | -0.3885 | -0.1161 | 2.0591 |
| 2.9492 | -0.0983 | 0.3153 | 0.6839 | -0.5372 | 1.3697 | -1.2616 | 0.8833 | -0.2837 | 1.746 | -0.0384 | 0.5035 | 1.6564 | 1.6464 | -1.1044 | 3.2174 | 0.0277 | 0.4657 | 0.1153 | -0.817 | 0.7098 |
| 1.0593 | 0.4919 | -0.4985 | 1.8533 | -0.487 | 2.4775 | -0.1784 | 1.3063 | 0.9147 | -1.5746 | 0.7888 | 0.2224 | -0.165 | 1.1818 | -0.3283 | 1.057 | -0.0928 | -0.4543 | 1.1822 | -0.1749 | 2.3466 |
| 0.2055 | 0.7592 | 1.0722 | -1.1083 | 0.5119 | -0.1066 | 0.6218 | -2.2714 | 0.7561 | 1.5679 | -0.3563 | 2.2188 | 1.9975 | 1.1686 | -2.1826 | -0.2037 | -0.4653 | 0.0479 | -1.113 | -0.1268 | -0.38 |
| 0.9321 | -2.4603 | -2.3683 | 0.5845 | -0.126 | -1.132 | 0.2465 | 2.4433 | -0.5195 | 2.4869 | 1.4158 | -0.3649 | -1.3177 | 0.5295 | 3.1453 | -0.156 | -0.9841 | -1.0384 | -0.4807 | -0.4864 | -0.6418 |
| -2.7362 | -0.8662 | -0.596 | 1.1021 | 1.4125 | 2.8826 | 0.1588 | 2.6925 | 0.0512 | 0.5218 | -0.9016 | 2.7 | 4.1398 | -1.2855 | -0.342 | -1.5551 | -0.5485 | -1.0258 | 0.1749 | 1.7176 | 1.4372 |
| -0.0085 | 0.6838 | 2.532 | 1.4447 | 1.4559 | 2.5567 | 0.2328 | 0.4783 | -0.1387 | 1.4629 | 2.9162 | -1.9238 | -0.2557 | -0.115 | 0.014 | 0.0178 | 0.1252 | 2.9812 | 1.4722 | 0.9165 | 1.4229 |
| 0.6981 | 2.3177 | 3.5524 | 2.7081 | 0.7654 | 0.0053 | 0.3786 | 0.1118 | 2.1312 | -1.7982 | -0.8967 | -1.4099 | -0.7532 | 0.2164 | 0.0984 | 1.5909 | 2.8542 | 2.3117 | 2.1544 | 2.117 | -0.7283 |
| 1.7749 | 3.3676 | -0.2558 | 0.4238 | 0.7355 | 0.1225 | 0.8694 | -0.9606 | 0.3301 | -0.6443 | 0.4729 | 0.3911 | 0.3216 | 1.7377 | 1.2345 | 0.7491 | 2.4833 | -0.5332 | 0.025 | 1.5605 | 0.9212 |
| 0.6576 | 0.2836 | 0.8631 | -0.4549 | 2.2926 | -1.0515 | 1.1495 | 0.1717 | 1.3394 | 1.1066 | 0.2698 | 1.7883 | -0.1611 | -0.3671 | 1.6004 | 0.0835 | 0.2823 | 0.4154 | -1.4077 | 1.9031 | -1.1747 |
| 1.0921 | -0.397 | 1.3018 | 0.482 | 2.8271 | 0.2997 | 0.9624 | 1.2266 | -1.9306 | 0.8444 | 0.844 | -0.485 | 2.2968 | -0.2499 | 1.5754 | 0.5945 | -0.3748 | 0.0563 | -0.7973 | 1.8568 | 0.703 |
| 0.072 | 0.7601 | -1.6368 | -0.6963 | -0.4502 | -0.6725 | -2.0379 | 0.5229 | 2.2579 | -0.1046 | 1.8361 | 1.3476 | -0.0738 | -1.6483 | -0.6327 | -0.5097 | 0.5452 | -0.1231 | -0.9216 | -0.4417 | -0.4451 |
| -1.95 | -2.4122 | -0.8109 | -0.7954 | 0.1296 | 0.325 | 2.4242 | -1.5994 | 2.1476 | 0.5519 | -1.2837 | -0.4866 | 2.0042 | 3.6084 | -1.2019 | -0.7687 | -1.0184 | -1.1441 | -1.0997 | 0.1574 | 1.9274 |
| -0.4633 | 0.7221 | 0.6876 | 1.5949 | 1.0497 | 0.8779 | 2.8104 | -0.3849 | 0.215 | -0.228 | 2.4501 | 4.3381 | -1.795 | 0.3639 | 0.5213 | -0.3557 | -0.0262 | -0.0098 | 2.0673 | 0.5154 | -0.3781 |
| 0.7888 | 1.8595 | 2.3428 | 1.4648 | 2.1653 | 1.6972 | 0.7161 | 0.1551 | 0.8021 | 3.0556 | -1.9491 | 0.2849 | -0.4329 | -0.0667 | 0.2402 | 0.8339 | 2.3367 | 2.9364 | 1.1473 | 1.1542 | 1.2157 |
|  |  |  |  |  |  |  |  |  |  |  |  |  |  |  |  |  |  |  |  |  |
|  |  |  |  |  |  |  |  |  |  |  |  |  |  |  |  |  |  |  |  |  |
| = |  |  |  |  |  |  |  |  |  |  |  |  |  |  |  |  |  |  |  |  |
|  |  |  |  |  |  |  |  |  |  |  |  |  |  |  |  |  |  |  |  |  |
| 1.0499 | 1.6601 | 1.8105 | -1.9679 | 1.5153 | -2.7824 | 1.3112 | -0.602 | -1.1975 | 0.3599 | 1.644 | 1.4835 | 2.479 | 0.2327 | -0.4767 | 2.0626 | 1.5023 | 1.3913 | -2.2746 | 1.2923 | -1.8795 |
| 1.1574 | -1.7469 | 0.3922 | -0.831 | 1.4678 | -0.8518 | -0.0987 | 1.2574 | 1.7481 | 0.8943 | 1.5467 | 0.0115 | 1.486 | 0.5917 | -0.8116 | 0.5839 | -1.8078 | 1.0775 | 1.1303 | 2.3255 | -0.5901 |
| 2.1347 | 0.6601 | 1.3008 | -0.1053 | 1.6672 | 1.4565 | 1.1586 | 0.8152 | 2.9543 | -0.3485 | 1.5601 | -0.7068 | -0.3854 | 1.8758 | -1.4264 | -0.3191 | 2.6753 | 2.1358 | 0.7322 | 0.8512 | 0.8071 |
| -0.0576 | 0.5899 | -1.9407 | 0.1173 | -0.1423 | 0.8959 | 2.6011 | 1.9788 | 1.9337 | -0.2301 | 0.4854 | -0.0372 | -0.3371 | 1.6554 | 1.9465 | 1.1166 | 0.4746 | -1.7346 | 0.1152 | -0.8599 | -0.1821 |
| -1.4543 | 0.5303 | 3.4587 | -0.4766 | 1.919 | 2.6939 | -0.5297 | -0.6716 | -0.6449 | -1.9102 | -0.518 | 0.0058 | 0.2375 | 0.2362 | -0.2092 | -1.2093 | 0.2519 | 2.1147 | -0.3105 | 1.7996 | 1.2629 |
| 2.1632 | -1.5858 | 2.5074 | 0.1846 | -0.9013 | 0.6582 | 1.261 | 3.6024 | -0.8489 | 0.1144 | -0.3339 | -0.9494 | -0.7753 | 0.1989 | 2.8332 | 1.5678 | -0.7401 | 2.4204 | 0.6063 | -1.1761 | -0.1346 |
| 1.1593 | -0.2367 | -0.1083 | 0.811 | 2.3672 | 3.8136 | -1.4121 | -0.2571 | -0.5458 | 0.0852 | -0.3225 | 0.6285 | 2.101 | 1.0658 | 0.1319 | 0.8261 | 0.5016 | -0.6218 | 0.3533 | 2.1855 | 2.9989 |
| -0.2587 | 1.0573 | 1.8001 | 2.5881 | -1.8121 | 1.4237 | -1.453 | 0.8102 | -0.0261 | 0.3861 | 0.8539 | 2.8631 | -0.7304 | 1.2762 | -0.0519 | 0.3539 | 1.2856 | 1.782 | 2.5904 | -2.2187 | 1.204 |
| 1.4182 | 1.5349 | -2.0742 | 0.7204 | -2.231 | 1.1449 | -1.1184 | -1.1964 | 1.7338 | 0.9154 | 1.3856 | 1.3911 | -0.6508 | 0.1413 | 2.8862 | 2.1131 | 0.8416 | -1.7564 | 1.084 | -0.7984 | 2.2191 |
| -1.6129 | 0.8135 | -0.9429 | 1.7653 | -0.3394 | 1.2672 | 1.8435 | 2.7589 | 0.9654 | 1.9763 | -0.0634 | 1.3046 | -0.2551 | -0.9482 | 1.2018 | -1.5399 | 0.9826 | 0.8539 | 2.1032 | 0.3221 | 0.8971 |
| 0.1768 | 1.1148 | 0.2894 | -1.0581 | 1.3759 | 1.5215 | 0.7477 | 2.5716 | 0.4785 | 1.508 | -0.6369 | 0.5818 | 1.531 | -1.1552 | 1.6323 | 1.1404 | 1.8052 | 0.5896 | -1.221 | 0.974 | -0.2908 |
| 0.6616 | -2.2081 | 0.4295 | 1.8311 | 1.3829 | 3.3954 | 2.762 | 1.272 | -1.0248 | 0.573 | -0.293 | -0.1961 | 1.0136 | 1.2077 | 0.3282 | -0.2318 | -1.7568 | 0.3533 | 1.525 | 0.41 | 2.1935 |
| 0.7885 | 3.5988 | -0.6812 | 2.6206 | 2.8634 | -0.4866 | -0.6451 | -1.2272 | 2.2021 | -0.8713 | -0.775 | -0.1069 | -0.7764 | -0.343 | -0.5223 | 1.0275 | 2.5692 | -1.089 | 2.6804 | 1.8773 | -0.2581 |
| -1.2187 | 2.1827 | 0.2751 | -0.2231 | 0.2319 | 0.8188 | 3.9044 | -0.7868 | -1.2433 | -0.4939 | -1.2236 | -0.9228 | 0.9531 | 2.5286 | 1.0731 | -0.36 | 2.2091 | 0.6556 | -0.9797 | 0.2182 | 1.0963 |
| -0.2883 | -0.1923 | 0.7965 | 1.2735 | 3.7145 | -1.3121 | 0.1356 | -0.6421 | 0.7002 | -0.8149 | 0.3026 | 1.6838 | 2.0742 | 1.328 | 1.1478 | 0.7487 | -1.0614 | 0.8861 | 1.2446 | 2.9858 | -2.145 |
| 1.2751 | 1.5843 | 2.4034 | -1.7144 | 1.2925 | -1.9061 | -0.077 | -0.7484 | -0.4027 | 1.2023 | 2.2527 | 1.491 | 1.3027 | 0.0235 | -0.2601 | 1.1691 | 1.9962 | 2.1697 | -2.5079 | 1.183 | -1.3505 |
| 0.5558 | -1.8332 | 0.5232 | -1.0408 | 1.8953 | -0.8723 | -1.0488 | 0.8944 | 0.5128 | 0.937 | 2.103 | -0.2902 | 0.58 | 1.0853 | 0.9597 | 0.5644 | -1.6066 | 0.6475 | 0.2378 | 2.8026 | -0.3478 |
| 0.9843 | -0.1049 | 1.2426 | 0.1617 | 1.982 | 2.0477 | 1.1614 | 0.877 | 2.7607 | -0.2293 | 1.4119 | -0.1097 | -0.6605 | 2.1943 | -1.7955 | 0.6147 | 2.1094 | 2.75 | 1.0492 | 1.3107 | 1.0813 |
| 0.65 | 0.2834 | -1.6835 | 1.0518 | 0.3991 | 0.3181 | 2.6881 | -0.0138 | 1.9094 | -0.26 | -0.096 | 0.8236 | -1.0915 | 1.7418 | 2.1228 | 1.6573 | 0.3696 | -1.3217 | 0.5065 | -0.8366 | -0.2053 |
| -2.0653 | 0.4402 | 2.69 | 0.6729 | 1.8584 | 2.3696 | -0.1239 | -0.5411 | -0.5046 | -0.2906 | -0.5036 | 0.7724 | 0.9382 | -0.4138 | -0.2984 | -2.03 | 0.671 | 2.157 | 0.0571 | 2.4176 | 0.6003 |
| 2.9126 | -1.5829 | 2.3541 | 1.9104 | -1.008 | -0.352 | -0.5931 | 3.0308 | -0.9851 | -0.1968 | -0.7536 | -1.123 | -1.0046 | 0.7828 | 2.2227 | 2.5902 | -1.3376 | 2.3998 | 2.135 | -1.117 | -0.5938 |
| 2.7917 | -0.0957 | -0.2723 | 0.0355 | 1.2283 | 3.9186 | -1.513 | -0.2348 | -0.6387 | -0.6166 | -0.7999 | 0.4031 | 2.184 | 0.7086 | 0.3115 | 1.8649 | 0.4127 | -0.4368 | 0.9158 | 1.609 | 3.3173 |
| -0.4638 | 0.8056 | 1.7951 | 2.8791 | -1.5778 | 1.1869 | -1.3453 | 0.878 | -0.0366 | 0.4085 | 1.1889 | 2.4446 | 0.1524 | 1.0516 | 0.6306 | -1.0451 | 0.8892 | 1.4898 | 2.4772 | -2.0839 | 1.8444 |
|  |  |  |  |  |  |  |  |  |  |  |  |  |  |  |  |  |  |  |  |  |
|  |  |  |  |  |  |  |  |  |  |  |  |  |  |  |  |  |  |  |  |  |
| = |  |  |  |  |  |  |  |  |  |  |  |  |  |  |  |  |  |  |  |  |
|  |  |  |  |  |  |  |  |  |  |  |  |  |  |  |  |  |  |  |  |  |
| 2.529 | -1.2357 | 0.0526 | 0.347 | 0.7383 | 0.2401 | 1.6102 | -0.6612 | -1.2587 | 2.1733 | 1.0809 | -0.0935 | -1.7498 | 1.071 | -0.3126 | 2.9179 | -1.7456 | 1.7919 | 0.4472 | 2.7894 | 2.2514 |
| 0.2416 | 1.29 | 1.2219 | 0.7168 | 1.1073 | -1.0486 | 1.317 | 0.8332 | -0.7701 | -1.3057 | -1.2927 | 0.6286 | 3.0815 | 2.0742 | -1.3215 | 0.514 | 1.3095 | 0.892 | 1.4789 | 1.5019 | -1.7261 |
| 0.3051 | 0.984 | 2.8619 | -0.3454 | 2.0176 | -0.1746 | -0.219 | 0.4442 | -2.0532 | 1.2228 | 3.8551 | 1.9136 | 0.7079 | -0.9862 | 1.2377 | 0.2948 | -0.6444 | 1.0884 | -1.3867 | 2.9234 | -0.2811 |
| 2.443 | 0.5653 | 1.6029 | -0.3786 | -0.1845 | -0.4574 | -0.9946 | 1.135 | 1.5158 | 0.6616 | 0.9912 | -1.5575 | 0.3014 | -0.1064 | -0.7043 | 1.2636 | 0.8389 | 1.1975 | 0.208 | 0.5862 | -0.7655 |
| 0.3898 | -0.3176 | -0.5748 | -1.3741 | -0.5178 | -0.035 | 0.2612 | 0.1436 | -1.0295 | -1.6122 | 0.6338 | 3.6302 | -0.2621 | 1.6748 | 1.4775 | -0.5479 | 0.7309 | -1.1243 | -1.9418 | -1.1611 | 0.4777 |
| 0.3951 | 3.2972 | -1.4257 | -0.9836 | -0.6869 | -1.0017 | -1.3141 | -0.6597 | 1.4596 | 1.6938 | -0.2821 | 1.325 | 0.3617 | -0.5403 | 0.2099 | -0.9504 | 2.4324 | -1.5549 | -1.092 | -1.7021 | 0.1593 |
| -1.3637 | -0.61 | -1.6815 | -0.008 | -1.0517 | 1.4857 | 2.2203 | 1.9282 | 0.9623 | -0.2974 | -0.9337 | 0.7774 | 0.1208 | 0.0398 | 1.8507 | -0.6159 | -1.2439 | -1.5155 | 0.6029 | -2.0149 | 0.6825 |
| -1.7669 | 0.9224 | -1.5448 | -0.1399 | 0.6819 | 2.0196 | -0.1763 | 0.5356 | -0.1809 | 0.0851 | 0.9923 | 1.1595 | 0.7092 | -1.9207 | 0.124 | -0.4501 | 1.568 | -1.8917 | -0.5138 | -0.5315 | 2.3367 |
| -1.0104 | -0.1386 | 1.5091 | 0.2892 | 0.731 | 1.27 | -0.739 | -0.5738 | 1.825 | 0.9577 | -0.5076 | -1.4005 | 0.7919 | 0.3667 | 2.5762 | -2.1655 | 1.2665 | 1.4663 | 1.6565 | 1.8189 | 2.0274 |
| 1.3124 | 1.2783 | 0.9315 | 1.4404 | -0.7922 | 1.9501 | 0.1836 | -0.4893 | -0.1603 | -1.3623 | 0.6725 | 2.8942 | 1.5925 | -0.0639 | 0.6114 | 1.8673 | 0.5525 | -0.7046 | 1.2323 | -1.3101 | 1.688 |
| 0.4544 | 3.0827 | 0.4127 | 1.0299 | -1.0017 | -0.2979 | 0.4429 | -1.5787 | 1.068 | 2.1377 | 1.5519 | 1.0097 | -1.883 | 1.7592 | -0.996 | 0.2463 | 1.1203 | 0.9615 | 1.4827 | 0.1769 | 0.3147 |
| 0.4425 | 1.6076 | -0.2889 | 0.3231 | -1.085 | -0.4087 | 0.7952 | 0.9556 | 0.0384 | -0.164 | -1.5183 | 0.4745 | 1.9518 | -0.049 | 0.7278 | 0.9791 | 1.7045 | 0.2013 | 0.6933 | -1.3894 | -0.5589 |
| -0.6063 | -1.1326 | 2.342 | -0.2623 | -0.5778 | -0.0162 | -0.845 | -0.4267 | -1.0087 | 0.5432 | 3.7587 | 0.1162 | 2.3074 | 2.1028 | 0.2726 | -0.053 | -1.1224 | 1.5535 | -0.0674 | -0.1371 | -2.0613 |
| 2.9716 | -1.2298 | -0.873 | -0.7888 | -1.0205 | -1.4557 | 0.7102 | 2.2629 | 1.7765 | -0.9599 | 1.2462 | 0.1467 | 0.0476 | 0.3965 | -0.2573 | 2.4501 | -1.3595 | -1.3969 | -1.1994 | 0.0333 | -1.8109 |
| -0.5314 | -1.3008 | 0.8571 | -0.3672 | 0.8163 | 1.4748 | 2.2471 | 0.3885 | 0.6713 | -0.8789 | 0.0805 | 0.9079 | 1.0534 | 1.7021 | -1.0927 | -1.6936 | -1.1618 | 1.4933 | -1.0957 | 0.3483 | 0.6983 |
| 2.2173 | -2.2997 | -0.6494 | 0.4538 | 1.2377 | 0.3709 | 0.6502 | -0.0785 | -0.6571 | 1.6175 | 1.2272 | 0.2539 | -1.859 | 0.1904 | -0.436 | 2.5702 | -2.7438 | 0.5729 | 0.3837 | 2.0076 | 3.2165 |
| -0.7221 | 1.0927 | 1.3536 | 0.3735 | 1.28 | -1.4084 | 0.163 | 1.3117 | 0.9674 | -0.4595 | -1.2161 | 0.612 | 1.7651 | 1.6932 | -3.2039 | 0.4923 | 1.0773 | 1.6988 | 2.0004 | 1.6654 | -2.0998 |
| 0.4095 | 0.8473 | 2.4834 | -0.9569 | 1.8185 | -0.1342 | -0.15 | 0.2809 | -1.3969 | 0.4781 | 3.2517 | 1.9499 | 0.6376 | -0.3291 | 1.9614 | 0.091 | -0.9889 | 0.4897 | -2.5544 | 1.5669 | -0.036 |
| 2.277 | -0.1348 | 0.8564 | -0.9032 | 0.1587 | -0.4354 | -1.7841 | 0.8289 | 2.3211 | 1.6441 | 0.7936 | -1.9641 | 1.0346 | -0.4315 | -2.022 | 1.2301 | 0.3737 | 0.3891 | -0.1015 | 0.435 | -0.5732 |
| 0.0892 | -0.456 | 0.0457 | -0.394 | 0.0679 | 0.8311 | 0.8955 | -0.3202 | -0.6429 | -1.7981 | 0.6274 | 4.1874 | 0.139 | 1.2552 | 1.3404 | 0.2156 | 0.8385 | -1.5885 | -1.4003 | -0.9154 | 0.5862 |
| -0.011 | 2.8398 | -1.19 | 0.1985 | -0.7909 | -0.8368 | -0.4664 | -0.2955 | 2.175 | 3.5582 | -0.1406 | 1.987 | 0.5692 | -0.0468 | -0.5644 | -1.4925 | 1.9886 | -1.372 | -0.2798 | -2.1036 | 0.5062 |
| -1.0933 | -0.7539 | -1.0893 | -0.5495 | -1.0609 | 0.7087 | 1.4947 | 1.803 | 0.2102 | 1.4955 | -0.4949 | 0.5581 | 0.878 | -0.7803 | 2.1426 | -0.8347 | -1.0711 | -1.3969 | 0.1507 | -1.4503 | 0.5672 |
| -1.761 | 0.9976 | -0.9636 | 0.2924 | 0.6625 | 1.7581 | -0.4739 | 0.5706 | -0.4281 | -0.1142 | 0.6744 | 0.9194 | 1.2624 | -1.3877 | -0.3932 | -1.9166 | 1.3437 | -1.282 | -0.3023 | -0.5598 | 1.9779 |
|  |  |  |  |  |  |  |  |  |  |  |  |  |  |  |  |  |  |  |  |  |
|  |  |  |  |  |  |  |  |  |  |  |  |  |  |  |  |  |  |  |  |  |
| = |  |  |  |  |  |  |  |  |  |  |  |  |  |  |  |  |  |  |  |  |
|  |  |  |  |  |  |  |  |  |  |  |  |  |  |  |  |  |  |  |  |  |
| 1.4727 | -0.3972 | -1.0066 | 2.0171 | 1.5512 | 0.2652 | -0.9881 | 0.65 | 0.4092 | 1.635 | -0.9618 | 1.8456 | -0.043 | 1.6075 | 2.3029 | 2.2245 | 0.0672 | 0.066 | 0.6752 | 0.1381 | 2.1176 |
| 1.551 | 0.6198 | 0.3505 | -1.2882 | -0.5257 | -0.6648 | 2.4341 | 1.007 | -1.1146 | 0.8861 | 1.9784 | 2.9273 | 0.5562 | 2.0281 | -2.6185 | 1.3671 | 0.9542 | -0.6701 | 0.3824 | 0.3993 | -0.4933 |
| -0.066 | -0.0756 | -0.8412 | -0.1607 | 2.7073 | 2.1909 | 0.8575 | -0.2091 | 1.3825 | 0.6638 | -1.9939 | -0.7421 | -0.8622 | 1.4008 | 0.553 | -0.1287 | 0.7261 | 0.0202 | -1.3537 | 2.0968 | 2.2506 |
| -0.3431 | 0.6351 | 0.7608 | 0.9909 | 1.7415 | -1.7259 | 0.6302 | 0.3437 | -1.5637 | 1.6052 | 0.3844 | 1.6787 | 0.417 | 0.4582 | 0.1147 | -0.1894 | 0.0637 | -0.0044 | 0.8604 | 1.9972 | -1.5435 |
| -1.5933 | 0.2708 | -1.1359 | -2.3146 | 0.8212 | 3.6763 | -0.1352 | 2.6836 | 0.6066 | 0.0803 | 1.4462 | -1.8359 | -1.1731 | -1.5871 | -0.2018 | -3.0114 | 0.0062 | -0.8857 | -1.2814 | 1.1206 | 3.367 |
| -2.6745 | -1.1141 | 1.8608 | 1.7413 | -0.3444 | 0.3403 | -0.9303 | -0.3756 | -0.2008 | -0.988 | 0.5999 | -2.1086 | -1.4021 | -2.5073 | -1.2026 | -3.5621 | -1.3815 | 1.6848 | 3.0146 | 0.7694 | -0.2758 |
| 1.1745 | 1.6211 | 0.3572 | -0.4187 | -1.4547 | 0.0586 | -0.68 | -0.309 | 1.265 | -0.4672 | -1.1344 | -0.8446 | 0.3478 | -2.3637 | 1.0652 | 2.0292 | 1.5951 | 1.0528 | -1.4048 | -0.9717 | 1.2287 |
| 1.0376 | 0.6383 | 0.129 | -0.0048 | -0.3451 | 1.2036 | 1.0845 | -1.1574 | -0.8168 | 0.1427 | 1.6177 | -1.6072 | -1.0761 | 0.7043 | 0.8428 | 0.8274 | 0.7089 | -0.2056 | 1.1809 | -0.6357 | 0.326 |
| -1.0773 | -0.1406 | 1.5084 | 1.4028 | 0.4719 | -0.8699 | -0.4049 | 1.391 | 1.5587 | -0.8515 | 2.1054 | 1.8005 | 1.7544 | 1.9238 | 2.3101 | -1.194 | 1.2146 | 0.9847 | -1.5602 | 2.2731 | -0.5409 |
| 0.3815 | 0.0975 | -0.3762 | -0.8677 | -0.2129 | 1.8857 | 2.2335 | 0.3382 | 1.2855 | 1.3587 | 1.2981 | -1.3194 | 2.0923 | -1.4211 | 1.2725 | 1.216 | 0.0604 | 1.3882 | 0.8364 | -0.7571 | 1.9894 |
| 0.7352 | -1.1395 | -0.5383 | 1.5798 | 1.6551 | 0.8478 | -1.622 | 1.3554 | 1.1679 | -2.0183 | 0.1793 | 0.294 | 1.8307 | 0.4365 | 0.0583 | -0.2706 | -1.054 | -0.8909 | 0.4088 | 1.7678 | 1.2138 |
| 0.8067 | 0.0607 | 0.4565 | 0.8404 | -2.3025 | 0.8431 | 2.7869 | 1.2222 | 1.7473 | 0.1396 | 2.2329 | 1.8371 | 0.2393 | 0.0326 | -0.6022 | -0.0987 | -1.094 | 0.8665 | 0.9568 | -2.2154 | 1.0301 |
| 0.3055 | -1.3094 | -1.8296 | 1.2544 | 3.9518 | 1.1368 | 2.1077 | -0.0423 | 0.3031 | 0.2047 | -1.8334 | 0.5717 | -1.1491 | -0.1308 | -3.0417 | -0.3715 | -1.5136 | -1.1992 | 0.5686 | 4.962 | 0.5552 |
| -0.0175 | 1.7355 | 1.9411 | -0.3293 | 0.493 | -1.5617 | 0.0385 | -0.4567 | -0.5891 | 1.4339 | -1.4452 | -0.6179 | -2.5201 | -0.8479 | -3.0841 | -1.148 | 1.4404 | 3.2674 | 0.0958 | 0.0402 | -0.7232 |
| 1.3085 | 0.3469 | 0.1633 | -1.1897 | 0.1969 | -0.5798 | 1.0114 | 1.7169 | -1.1796 | -0.8867 | -1.5816 | 1.2719 | -1.7519 | 0.6867 | 0.9529 | 0.8192 | 0.1953 | 0.6329 | -1.314 | 1.5307 | -1.0649 |
| 1.0672 | -0.5012 | 0.0769 | 0.39 | 1.1636 | 0.6111 | -1.0513 | -0.725 | -0.0173 | 2.1945 | -1.4558 | 1.5032 | 0.7897 | 1.0822 | 2.5113 | 0.9316 | -0.3918 | 0.2451 | -0.6175 | 0.3707 | 2.1196 |
| 0.1897 | 1.0439 | 0.2253 | 0.0144 | -0.2354 | -0.7148 | 1.7313 | 1.6832 | -1.0873 | 0.8799 | 2.092 | 4.5438 | 1.5269 | 2.0885 | -2.4316 | 2.1802 | 1.3999 | -1.244 | 1.4076 | 0.1606 | -1.026 |
| 0.6685 | -0.0394 | -0.9242 | -0.3153 | 2.4713 | 1.9131 | 0.0031 | 0.5836 | 0.8221 | 1.4723 | -2.1954 | 0.0057 | -1.7214 | 1.4225 | 0.6098 | 0.6715 | 0.5804 | 0.2953 | -0.8527 | 2.153 | 1.8243 |
| -1.3713 | -0.1555 | 1.6702 | 1.2351 | 1.1533 | -2.318 | 1.1542 | 0.5246 | -1.731 | 1.0143 | -0.0066 | 2.1845 | 0.5639 | 0.3084 | 0.1844 | -0.9875 | -0.712 | 0.3823 | 1.8458 | 1.7137 | -1.831 |
| -1.4839 | -0.0711 | -0.6333 | -2.4425 | 0.6418 | 3.8443 | 0.6557 | 2.1943 | 0.1086 | 1.0008 | 1.5064 | -1.8217 | -0.0971 | -1.1461 | -0.447 | -2.2724 | 0.0664 | -0.4426 | -1.6476 | 0.5971 | 3.3813 |
| -1.5481 | -0.8989 | 2.301 | 3.7579 | 0.6183 | 1.0416 | 0.8377 | -0.2975 | 0.0807 | -1.3645 | 0.6068 | -1.8001 | -0.5054 | -2.5392 | -1.3032 | -1.7141 | -1.5804 | 0.9003 | 3.163 | 0.9096 | 0.2307 |
| 1.1774 | 1.3848 | 0.5804 | 1.0244 | -1.4559 | 0.5834 | -0.2222 | -0.9072 | 1.1927 | -1.0722 | -0.8007 | -1.934 | -0.1767 | -2.6921 | 0.4577 | 1.6856 | 2.2924 | 1.5725 | 0.2583 | -1.5035 | 1.1512 |
| 0.3578 | 0.4705 | 0.1016 | -0.1989 | -0.37 | 1.1956 | 1.3635 | -1.4873 | -0.2728 | -0.7284 | 1.408 | -2.1029 | -0.5212 | 0.7101 | 1.1235 | -0.7825 | 1.0618 | -0.6961 | 1.2165 | -0.4949 | 0.4634 |
|  |  |  |  |  |  |  |  |  |  |  |  |  |  |  |  |  |  |  |  |  |
|  |  |  |  |  |  |  |  |  |  |  |  |  |  |  |  |  |  |  |  |  |
| = |  |  |  |  |  |  |  |  |  |  |  |  |  |  |  |  |  |  |  |  |
|  |  |  |  |  |  |  |  |  |  |  |  |  |  |  |  |  |  |  |  |  |
| -1.5999 | -0.5033 | 0.1143 | 0.6582 | -0.8034 | 0.9803 | -0.3647 | 1.0927 | 2.1758 | 2.3907 | -0.0855 | 0.5937 | -0.7529 | 0.5705 | 1.9542 | -0.5064 | -1.4012 | -0.1899 | -0.7033 | -2.0255 | 0.9267 |
| 2.367 | 0.162 | -2.8515 | 1.8308 | 2.6502 | 1.7139 | 1.5927 | 2.7447 | -2.343 | 0.7937 | 1.6837 | -0.0946 | 0.5023 | 0.4531 | -1.2525 | 2.2245 | 0.2137 | -2.2603 | 1.8367 | 3.0879 | 1.1061 |
| 0.9277 | -1.1924 | 1.0727 | 0.9754 | -1.0967 | -0.6608 | -0.0285 | 1.7063 | 1.3304 | -0.3029 | -0.6872 | 0.5471 | -1.0133 | 2.3268 | 1.8861 | 0.7287 | 0.1342 | 1.5853 | 1.2504 | -0.3133 | 0.3691 |
| 1.259 | 1.3017 | -0.5409 | 1.0912 | 0.1165 | 1.6552 | 0.6792 | 0.3796 | 0.5108 | -0.1758 | -1.3496 | -0.209 | 0.47 | 1.9679 | -1.0642 | 1.8785 | 2.4215 | 0.3731 | 1.4466 | 0.7939 | 0.8094 |
| -0.0404 | 2.1839 | 0.7096 | 0.9819 | 0.7524 | -1.8 | -0.5278 | -1.6209 | -0.5092 | -2.239 | -0.8452 | -0.4221 | -1.5454 | 1.4947 | 3.6593 | 0.2547 | 1.1628 | 1.0475 | 0.5967 | -0.0032 | -1.6476 |
| -0.7399 | 0.0326 | 0.5189 | -0.8983 | 0.5692 | -1.0866 | -1.1157 | -3.2642 | -1.0561 | -3.364 | -0.7693 | 2.1571 | 2.3073 | 0.1786 | -0.9685 | -0.2635 | 0.3622 | -0.0255 | -2.1184 | 0.251 | -1.1065 |
| -1.0572 | -1.9302 | 1.6351 | 0.197 | -0.8293 | -0.4688 | -0.2045 | -2.2321 | 1.3653 | 1.0199 | 1.9798 | 0.5848 | -1.0005 | -1.2442 | 1.4832 | -0.4884 | 0.6724 | 1.0744 | 0.1161 | -0.3594 | -1.5255 |
| 2.6123 | -1.4515 | -1.2649 | -0.4788 | 0.5689 | -1.0884 | 0.2286 | 1.3898 | 0.3403 | -0.4246 | 0.3049 | -1.2635 | 1.3207 | -0.7503 | 0.7781 | 1.9799 | -0.6973 | -0.9612 | -0.5761 | -0.1317 | -1.4041 |
| -0.9203 | 0.7842 | -0.102 | -1.6385 | 1.7435 | 2.9165 | 2.0344 | 1.9559 | 3.477 | 0.0004 | 0.9274 | 0.5797 | -0.4371 | 1.846 | -0.2724 | -1.3127 | 0.6569 | -0.3236 | -1.3563 | 1.0186 | 3.5703 |
| 2.0556 | -2.4753 | 1.0271 | 1.3331 | 1.0147 | -1.2068 | 2.9368 | -0.9678 | 1.0341 | 2.674 | 0.5448 | 0.2227 | -0.0904 | -1.0983 | 2.7688 | 2.1502 | -1.2126 | 1.0688 | 1.8376 | 1.4004 | 0.2763 |
| -1.7055 | 0.629 | 1.0797 | -0.7964 | 0.0002 | 0.6709 | 1.4223 | 1.3033 | 0.3579 | -0.4622 | -0.0198 | -1.5157 | 1.0342 | 1.7225 | 0.9047 | 0.0005 | 1.2127 | 1.0951 | 0.291 | 0.6205 | 0.029 |
| 3.4409 | 0.2969 | 1.3145 | -0.6402 | 1.4728 | 0.7776 | 0.4956 | 0.2677 | -1.2015 | -0.1246 | -0.3824 | 0.1602 | 1.3623 | -1.5524 | 1.1779 | 4.6431 | 1.524 | 1.2261 | 0.2471 | 1.1106 | -0.1334 |
| 1.2855 | 0.8088 | 1.2077 | -0.6573 | -1.7663 | 0.3968 | -1.2915 | 0.1071 | -1.8459 | -0.2934 | -1.7864 | -1.6104 | 1.5856 | 2.7796 | 0.8318 | -0.089 | 2.1564 | 0.1945 | 0.2583 | -1.5449 | 0.6167 |
| 1.4069 | -0.0757 | -1.322 | 0.829 | -0.693 | -0.7757 | -3.1329 | -0.8491 | -2.2179 | 0.0721 | 1.3774 | 2.0389 | 0.3098 | -0.0266 | -0.8809 | 1.8259 | 0.2532 | -1.8009 | 0.0616 | -0.6745 | -1.3355 |
| 0.7699 | 2.3276 | -0.106 | -0.7963 | -0.8507 | 0.5713 | -1.2248 | 1.2011 | 0.5824 | 0.7495 | -0.1645 | 0.4811 | -1.5396 | 2.0029 | -0.5355 | 1.0595 | 0.7752 | -0.0187 | -0.1194 | -0.7215 | 0.7407 |
| -1.2562 | -1.6967 | -0.4348 | 0.7117 | -1.1809 | 0.8656 | 0.4726 | 0.178 | 2.1431 | 0.858 | 0.1897 | 0.79 | -1.1364 | 0.4553 | 1.7609 | -0.6954 | -0.8649 | 0.033 | -0.239 | -1.3714 | 0.9994 |
| 1.1754 | 0.1501 | -1.9403 | 1.8483 | 3.1925 | 2.0524 | 1.4189 | 2.8769 | -2.7922 | 0.8465 | 1.1112 | 0.0538 | 1.4318 | 0.0664 | -1.1797 | 1.2189 | -0.2984 | -1.7147 | 2.1091 | 3.0186 | 1.1108 |
| -0.6358 | 0.5491 | 0.8064 | 0.706 | -1.3184 | 0.248 | -0.9628 | 1.6258 | 0.9798 | -0.2621 | -0.2976 | 0.6311 | -1.1296 | 2.0544 | 1.9249 | 0.0448 | 0.8378 | 1.1271 | 1.1541 | 0.1584 | -0.0543 |
| 0.2376 | -0.0349 | -0.8438 | -0.2428 | 0.0347 | 1.4392 | 0.8217 | 0.4452 | -0.0783 | -0.8055 | -1.7085 | 0.2201 | 1.5728 | 1.867 | -1.033 | 0.5454 | 1 | 0.1684 | 1.3773 | 0.1824 | 0.6003 |
| 1.3474 | 1.1148 | 0.1279 | 0.7239 | 1.1125 | -2.0282 | 0.3626 | -1.0641 | -0.9173 | -0.8967 | -0.8254 | -0.4971 | -1.3352 | 1.3272 | 4.8553 | 1.6796 | 0.7941 | 0.3074 | 0.3833 | 1.1413 | -1.6523 |
| 0.4122 | 1.1659 | 0.3577 | -0.3775 | 0.5731 | -2.0289 | -1.0265 | -2.2334 | -1.7263 | -1.2599 | -1.5766 | 1.9271 | 3.2549 | 0.9612 | -0.7849 | 1.2294 | 0.1872 | 0.8337 | -0.8475 | 0.4836 | -1.2912 |
| -0.0721 | -1.6693 | 1.6611 | -0.1514 | -0.3141 | -1.2698 | -0.4587 | -1.9379 | 0.9787 | 0.9805 | 1.941 | 0.854 | 0.2593 | -1.6493 | 1.875 | 0.2271 | 0.3275 | 0.3693 | 0.0456 | -0.5801 | -1.5834 |
| 2.6679 | -0.501 | -0.7899 | -0.6173 | 0.6716 | -1.6172 | 0.3447 | 1.0999 | 0.4363 | -0.367 | 0.826 | -1.2236 | 1.6575 | -0.3681 | 0.9903 | 1.978 | -0.086 | -0.5946 | -1.3239 | 0.1001 | -1.7762 |
|  |  |  |  |  |  |  |  |  |  |  |  |  |  |  |  |  |  |  |  |  |
|  |  |  |  |  |  |  |  |  |  |  |  |  |  |  |  |  |  |  |  |  |
| = |  |  |  |  |  |  |  |  |  |  |  |  |  |  |  |  |  |  |  |  |
|  |  |  |  |  |  |  |  |  |  |  |  |  |  |  |  |  |  |  |  |  |
| -1.2421 | -0.6741 | 1.6864 | 2.0341 | 0.1006 | 0.1466 | -0.2923 | 0.5592 | 1.6981 | -0.3192 | -0.1979 | -0.8907 | -0.0837 | -2.2825 | -0.2252 | -1.5148 | -4.0524 | 1.4626 | 1.9508 | -0.0469 | 2.2075 |
| 1.7235 | 0.9566 | -0.1914 | 0.6874 | 0.8504 | 0.3901 | 0.6068 | 0.074 | -2.0435 | 2.2125 | 0.808 | -1.7751 | 0.3002 | 3.41 | 0.6032 | 2.5964 | 0.567 | -1.5218 | 0.655 | 0.855 | 0.5083 |
| -0.0574 | 1.8964 | 1.1272 | 0.0155 | -0.8924 | 0.243 | -1.4497 | 1.8921 | 1.2113 | 0.2414 | 0.3777 | 2.46 | 1.18 | -0.2999 | 1.0541 | -0.3684 | 0.6867 | -0.1909 | -0.8319 | 0.0831 | -0.1337 |
| 0.5041 | 0.1425 | -0.227 | 0.0645 | -1.936 | 0.7918 | 0.1124 | 0.2734 | -0.3697 | 1.7655 | 2.1826 | 0.86 | 0.7315 | -0.1623 | 0.5608 | -0.2541 | -0.0312 | -0.721 | -0.553 | -1.4501 | 1.9016 |
| -0.3523 | -1.535 | 0.0451 | -1.5522 | -0.1849 | -2.0224 | -0.7169 | 1.253 | 1.1434 | 0.1431 | -0.0288 | 0.1147 | 0.2416 | -0.1471 | -1.3562 | -1.536 | -1.0294 | -0.6471 | -0.8314 | 0.3682 | -1.9782 |
| 0.2918 | -3.7354 | -1.2638 | -2.2239 | -0.3116 | 1.7388 | 0.52 | 0.8133 | -0.0962 | -0.1073 | 1.9893 | 0.7775 | -0.9774 | 1.3479 | -1.7751 | 0.9875 | -2.5952 | -1.4792 | -1.6874 | 0.7304 | 2.9661 |
| -0.1672 | -1.7507 | 0.9263 | 2.0326 | 0.3871 | 0.9713 | -0.0104 | -0.94 | 1.82 | 0.2287 | 0.1805 | 0.3384 | -0.2684 | -0.6747 | -0.501 | -0.9138 | -1.7571 | 1.2528 | 2.8216 | 0.8483 | 0.7938 |
| 0.6559 | 1.2313 | -0.9516 | -1.1562 | 0.5407 | -0.8638 | 1.1579 | -0.5682 | 0.794 | 1.1373 | -0.9756 | 0.0327 | -0.4613 | 0.4772 | -1.2535 | 0.9307 | 0.8995 | -1.5406 | -1.923 | 0.3413 | -0.7678 |
| 0.4038 | 2.2098 | 2.6291 | -0.615 | 0.5544 | 0.4866 | -1.2018 | 1.4193 | -0.4191 | -1.4621 | -0.6349 | -0.5181 | -1.8319 | 0.2245 | 4.4583 | -1.246 | 2.3204 | 1.8703 | 0.5999 | 1.6311 | 0.8412 |
| 1.8937 | -0.3672 | 1.6085 | 1.6927 | 0.7197 | 0.4674 | -0.3355 | -1.9557 | 2.1383 | 1.5383 | -1.0925 | 0.0139 | 2.6448 | 0.2154 | -0.4994 | 1.384 | -1.0055 | 1.05 | 2.0492 | 0.8315 | -0.1528 |
| 2.2164 | 0.8968 | 0.4746 | -0.0711 | 0.4861 | -2.445 | 0.6628 | 0.7414 | 0.1513 | 0.3705 | 2.3393 | 0.8787 | 1.1996 | 0.524 | -0.4751 | 1.7864 | -0.4614 | -0.4931 | 0.1865 | -0.7504 | -2.7953 |
| -0.4354 | 0.1895 | -0.9261 | 0.0433 | -0.1932 | 0.3146 | -0.292 | -0.4345 | 0.8658 | 4.2991 | 1.1138 | 1.333 | -0.1085 | 0.7992 | 0.7421 | 0.627 | -0.5893 | -1.443 | -1.9414 | 1.5137 | 0.8514 |
| -2.0328 | 0.6438 | -2.2207 | 0.0879 | -2.3031 | -0.9011 | 1.7093 | 1.4002 | 0.45 | -0.3136 | 0.7029 | 0.0177 | -0.0134 | -1.201 | 1.5131 | -1.4364 | 0.0818 | -1.4794 | 0.382 | -1.0549 | 0.1187 |
| -2.8818 | -1.1755 | -1.7102 | 0.0323 | 1.3632 | 0.8449 | 0.6326 | 0.6806 | -0.2922 | 2.674 | 0.7803 | -1.0063 | 1.0719 | -1.1548 | -2.2609 | -2.1558 | -1.0215 | -1.159 | 0.9589 | 2.8184 | 0.1666 |
| -1.5108 | 0.5507 | 1.6032 | 0.1982 | -0.6616 | 1.1864 | -0.7208 | 2.2534 | -0.57 | 0.7767 | 0.8055 | 0.1986 | -0.4546 | -0.5712 | 1.1678 | -1.7264 | 0.3933 | 1.5394 | 0.8297 | -0.7077 | 1.3184 |
| -0.3457 | -1.4616 | 2.3988 | 0.4461 | -0.3197 | -0.2359 | -0.8645 | 0.7398 | 1.1846 | -0.3631 | 0.0852 | -0.2758 | 0.0942 | -1.5617 | 0.5825 | -0.6958 | -2.8592 | 1.8719 | 0.6055 | -0.8343 | 1.5662 |
| 1.3904 | 1.372 | -1.4085 | 0.5871 | 0.5212 | -0.185 | 1.0301 | 0.0833 | -1.8846 | 1.1762 | -0.6098 | -1.3702 | 1.0802 | 3.7483 | 0.8264 | 2.2171 | 1.4114 | -0.1819 | 1.575 | 0.4527 | 0.0218 |
| -0.864 | 1.6931 | 0.9373 | 0.252 | -0.3079 | 0.4449 | -1.6287 | 2.6972 | 1.4888 | -0.0791 | 0.5202 | 2.3611 | 1.3967 | -0.332 | 1.0127 | -0.8075 | 0.7772 | 0.4627 | 0.383 | -1.1226 | -0.5968 |
| 0.7792 | 0.8445 | -0.6499 | -0.0452 | -2.0662 | 0.0148 | 1.6824 | 0.4005 | -0.1314 | 1.5763 | 1.3045 | 0.0249 | 1.3091 | 0.0297 | 1.2402 | -0.4277 | -0.102 | -0.0579 | -0.6254 | -1.7755 | 1.5212 |
| 0.0488 | -1.0662 | -0.1307 | -0.2519 | -0.035 | -1.7079 | -0.5993 | 1.4004 | 2.9828 | 1.3314 | -0.0894 | 0.0608 | 0.0514 | -0.0455 | -1.3108 | -0.7606 | -0.7215 | -1.3218 | 1.0508 | 0.3677 | -2.2247 |
| 0.3722 | -3.1116 | -1.703 | -1.8844 | -0.4545 | 1.515 | 1.9813 | 0.18 | -0.814 | 1.1663 | 0.8356 | 0.6621 | -0.5227 | 1.2281 | -1.6262 | 0.4107 | -2.1151 | -1.9409 | -1.1935 | 0.2519 | 2.469 |
| -0.8382 | -1.2194 | 1.301 | 0.5921 | 0.5939 | 1.2047 | 0.1696 | -0.7082 | 2.2876 | 1.3907 | -0.1174 | 0.2568 | -0.4849 | -0.856 | -1.0953 | -1.4851 | -0.9195 | 1.5536 | 2.436 | 0.5173 | 1.1531 |
| -0.0348 | 1.9579 | -0.8572 | -1.0193 | 1.8421 | -0.5238 | 1.9933 | 0.1446 | 0.8259 | 0.9171 | 0.0287 | 0.2306 | -0.2678 | 0.0046 | -1.0783 | 0.6297 | 1.4063 | -0.3699 | -0.4935 | 1.9953 | -1.4426 |
|  |  |  |  |  |  |  |  |  |  |  |  |  |  |  |  |  |  |  |  |  |
|  |  |  |  |  |  |  |  |  |  |  |  |  |  |  |  |  |  |  |  |  |
| = |  |  |  |  |  |  |  |  |  |  |  |  |  |  |  |  |  |  |  |  |
|  |  |  |  |  |  |  |  |  |  |  |  |  |  |  |  |  |  |  |  |  |
| -0.6561 | -0.2067 | 1.1278 | -1.9673 | -0.6292 | -0.6078 | 0.2938 | -2.0228 | -0.6452 | -1.0691 | -2.3031 | 1.8436 | 1.1606 | -0.3478 | 1.9476 | 0.9694 | 0.641 | -0.4021 | -2.4358 | 0.2921 | -1.9895 |
| 0.1649 | -0.6914 | -1.0287 | 2.499 | 0.9603 | -0.8664 | -0.3981 | 2.3305 | 0.09 | 3.0573 | 0.305 | -0.3437 | 0.8566 | 1.573 | -0.2172 | 0.4354 | -1.9551 | -1.0449 | 1.3399 | 0.988 | -0.7096 |
| -2.0958 | 2.4436 | 1.5567 | 0.7469 | 1.7684 | 1.5968 | 1.2672 | -0.5984 | 1.9772 | -0.8479 | 0.8916 | 0.3298 | -0.8029 | 0.5702 | -0.6308 | -3.0311 | 1.1152 | 1.8246 | -0.1941 | 2.2778 | 2.3704 |
| 0.072 | 0.1014 | 0.4234 | 1.5245 | 1.5518 | -0.1621 | 1.0215 | -0.9293 | 1.084 | -0.3887 | 0.8049 | -0.7417 | -0.6276 | -0.7393 | 0.0747 | 0.839 | -0.284 | 1.2443 | 1.3234 | 0.8208 | -1.995 |
| -0.0601 | 1.9169 | 0.1175 | -1.2823 | 0.0641 | -1.2961 | 0.5125 | -0.1262 | 0.4062 | -1.0243 | -0.7092 | -1.229 | -1.9217 | 0.7073 | -1.298 | 0.4924 | 2.0077 | -1.7744 | -2.0698 | 0.2634 | -2.1862 |
| -0.4237 | 0.4059 | 1.715 | -0.7341 | 2.8404 | 0.7472 | -0.2446 | 0.9816 | -1.7058 | 0.4189 | -2.2972 | -0.9194 | -1.4694 | -0.2517 | 2.117 | -1.7547 | 0.031 | 1.4389 | -1.0452 | 1.3601 | 1.2984 |
| 1.2036 | -1.1354 | 3.0435 | 0.8771 | 0.8579 | 0.5706 | -0.4612 | -1.1957 | -0.1194 | -1.3103 | -1.2144 | 1.4008 | 2.012 | 0.6469 | 0.218 | 2.5384 | -2.2571 | 2.0336 | 1.4068 | 0.7552 | 0.8347 |
| 2.2 | 0.3635 | 0.4798 | 0.9803 | -1.8728 | 0.0315 | -0.1614 | 1.1107 | -1.1522 | 0.9841 | 0.3533 | -1.6488 | -0.7326 | 0.7751 | -1.6383 | 1.8002 | 0.8406 | 1.0199 | 0.318 | -1.9088 | 0.7316 |
| 0.111 | 0.8454 | -2.0379 | -0.6736 | -0.4791 | 0.4089 | -1.6296 | -0.6877 | 4.1104 | -1.6037 | 3.1346 | 0.1722 | 0.1664 | 1.2602 | 1.3592 | 0.3911 | -0.3062 | -2.622 | -0.4323 | -0.7052 | 0.9944 |
| -0.6085 | -1.6891 | 2.6252 | 1.7769 | -0.2597 | -0.0609 | 1.8644 | 0.4543 | -1.2999 | -0.2304 | -0.637 | 0.8491 | 1.5874 | 0.2801 | -0.9912 | -1.7885 | -1.3394 | 1.6366 | 1.9706 | -0.7862 | 0.356 |
| 0.9702 | 1.223 | 0.5879 | 0.9773 | 1.4359 | 0.9829 | 0.166 | 1.3959 | 0.508 | 2.2526 | 0.0413 | -0.1417 | 0.155 | -0.6849 | -1.8626 | 0.3054 | 1.1919 | -1.0302 | 1.3522 | 1.4825 | 1.776 |
| -1.941 | 0.4288 | 1.5882 | 1.5869 | 0.1588 | 0.9031 | -1.8872 | 0.4967 | 1.0743 | 1.2701 | -0.7188 | -1.3866 | -0.903 | 1.066 | 1.2765 | -0.7996 | 0.7442 | 1.5775 | 0.3942 | -1.2453 | 1.0481 |
| 2.3672 | -0.05 | -0.7954 | 0.1201 | -1.0799 | 0.5008 | 1.3895 | 0.5578 | 1.697 | -1.0431 | -1.4635 | -1.9553 | 0.5605 | -1.112 | -0.0467 | 2.0687 | -1.2991 | -1.9208 | 1.1497 | -1.566 | 0.3079 |
| 0.3694 | 2.0543 | -0.8453 | 3.7373 | 1.0856 | 0.6299 | 0.436 | -1.132 | -1.8318 | -1.995 | -0.8881 | -1.0307 | 0.08 | 2.6391 | -0.7184 | -0.3083 | 1.6681 | -1.7656 | 1.7077 | 1.9236 | 1.058 |
| -1.3984 | 3.4547 | 0.2418 | 0.9548 | 0.6673 | -0.5954 | -0.2472 | -0.4216 | -0.3605 | -2.3262 | 0.4746 | 1.9219 | 0.4659 | -1.0329 | 1.6311 | -2.0763 | 1.9827 | 1.2144 | 0.9486 | 0.9107 | -1.2869 |
| -0.1502 | 0.267 | 1.1019 | -1.7042 | 0.8311 | 0.0044 | 0.1516 | -2.1279 | 0.2623 | -0.4542 | -1.8445 | 1.4534 | 1.1447 | -1.5727 | 1.1924 | 0.6823 | 0.5353 | 0.215 | -2.129 | 0.8471 | -0.8814 |
| -0.1253 | -1.3498 | -0.6302 | 2.1097 | 0.5245 | -1.26 | -0.788 | 3.5298 | 0.2589 | 3.2791 | 0.2348 | -0.0523 | 1.5368 | 1.9528 | 0.1452 | -0.9708 | -2.1058 | -0.4779 | 0.965 | 0.8461 | -0.8148 |
| -1.3847 | 3.5324 | 1.6885 | 0.7705 | 0.6633 | 2.0412 | 1.1332 | -0.6296 | 1.6604 | -1.2735 | 0.4718 | -0.2786 | -0.3618 | -0.8854 | -2.1014 | -1.7965 | 2.0849 | 1.6983 | 0.48 | 1.3825 | 2.3456 |
| 1.5227 | 0.6684 | 1.1732 | 1.7737 | 1.0819 | -0.238 | 1.4724 | 0.3519 | 0.9246 | -0.5046 | 0.2144 | -0.471 | -0.857 | -1.6537 | 0.3223 | 1.1219 | -0.408 | 1.8451 | 1.0784 | 0.6953 | -1.2696 |
| 0.2776 | 2.0186 | 1.9896 | 0.0069 | -0.5312 | -1.9118 | -0.0516 | 0.6018 | -0.1572 | -1.4931 | -0.7214 | -1.2011 | 0.3328 | 0.6416 | -0.6511 | 0.955 | 2.1099 | 0.1974 | -1.184 | -0.2655 | -2.0574 |
| -0.3342 | -0.7835 | 0.3936 | -0.2304 | 1.8086 | 0.3272 | 0.8907 | 1.3277 | -1.6053 | -0.4927 | -2.3487 | -1.3227 | -1.1154 | -0.414 | 2.0501 | -1.865 | -0.9132 | 1.1702 | -1.8345 | 1.2378 | 0.9874 |
| 2.0742 | -0.9421 | 3.5168 | 1.8476 | 1.1897 | -0.2952 | -0.6585 | -1.2569 | -0.6647 | -1.0988 | -0.9021 | 0.939 | 2.1266 | 0.041 | 0.4933 | 2.4907 | -2.057 | 2.438 | 2.2375 | 0.4428 | -0.371 |
| 3.5064 | 0.6748 | 0.6128 | 0.0976 | -1.0059 | -0.0183 | -0.3364 | -0.4775 | -1.2281 | 0.4701 | 1.2307 | -1.1327 | -0.5931 | 1.8207 | -2.1092 | 2.2003 | 1.2401 | 0.8794 | 0.5625 | -1.8423 | -0.2176 |
|  |  |  |  |  |  |  |  |  |  |  |  |  |  |  |  |  |  |  |  |  |
|  |  |  |  |  |  |  |  |  |  |  |  |  |  |  |  |  |  |  |  |  |
| = |  |  |  |  |  |  |  |  |  |  |  |  |  |  |  |  |  |  |  |  |
|  |  |  |  |  |  |  |  |  |  |  |  |  |  |  |  |  |  |  |  |  |
| 1.065 | -2.0609 | -1.6379 | 0.2593 | -3.408 | 2.3431 | 0.6447 | -2.2758 | 1.5585 | 1.4318 | 0.4083 | 0.5492 | -1.9677 | 2.7597 | -3.3035 | 1.2834 | -1.4186 | -1.6732 | 0.8717 | -1.0845 | 2.7139 |
| -0.9237 | 3.1073 | -0.0331 | 3.6541 | 0.4342 | -1.501 | 0.4081 | 2.5275 | -0.2278 | 0.1019 | -1.4591 | -0.5238 | 0.8814 | 1.718 | -0.7354 | -1.1417 | 3.4312 | 1.4707 | 4.174 | 0.5918 | -0.7865 |
| 1.3862 | -1.8023 | 1.5778 | -0.9446 | 0.6297 | -2.5191 | -1.0596 | 1.0877 | -1.1208 | -0.7289 | 0.7254 | 2.5342 | -0.1535 | 0.9322 | 4.2258 | -0.2687 | -0.823 | 1.2435 | -0.6087 | 2.2516 | -1.8292 |
| 1.2822 | -0.9238 | 1.4438 | -0.2541 | 0.526 | -0.4229 | -1.357 | -1.015 | 0.0214 | 0.5568 | -0.3172 | 0.2225 | 2.1359 | 0.715 | -1.6586 | 1.1189 | -0.5354 | 2.1587 | -0.8357 | 2.0264 | -0.4971 |
| 1.5137 | 1.0471 | 0.4139 | -1.1031 | -0.6159 | -1.4298 | -2.2062 | 0.2208 | -1.2545 | -0.2235 | 2.6299 | -0.1841 | -2.1196 | 0.4981 | -2.1638 | 0.6423 | 0.9754 | 0.5564 | -1.6432 | -1.3749 | -1.8036 |
| 0.5544 | 1.2167 | -1.7839 | -0.9955 | -2.5109 | -1.6038 | -2.0664 | -0.5403 | 2.6148 | -2.448 | -1.1898 | 1.5129 | -1.3002 | 0.1472 | 1.4669 | 0.1268 | 0.4594 | -0.9282 | -0.9736 | -1.495 | -1.0581 |
| -1.2557 | -1.0895 | 0.6318 | -0.6299 | -1.0255 | 0.9307 | 2.9497 | -0.4126 | 0.2824 | 1.7047 | -1.7328 | 1.1043 | 1.4096 | 1.1036 | 0.7469 | -0.6705 | -0.5767 | 1.0425 | -1.4415 | -0.8447 | 1.0824 |
| 0.0243 | 0.0477 | -0.419 | 0.6333 | 0.5378 | -2.4761 | 0.4783 | 1.2325 | -2.8255 | 1.3306 | 0.8058 | 1.0801 | 1.0158 | -1.7709 | 1.0633 | -0.6432 | 0.8451 | 0.2789 | 0.7168 | 1.4034 | -1.7438 |
| -1.5814 | -1.0629 | 2.446 | -1.8556 | 3.4474 | 0.2177 | -2.2742 | 1.2843 | 1.6657 | -0.9945 | -0.7056 | -2.2365 | 1.0081 | -1.2366 | 1.7375 | -1.6304 | -1.7443 | 3.1278 | -1.2322 | 3.4402 | 0.2837 |
| 2.8892 | -0.2737 | -2.5194 | 1.3033 | -0.6931 | 0.7786 | 1.8947 | 0.3302 | 0.2012 | -2.234 | -1.0993 | 1.1773 | 2.3957 | -1.3941 | -1.0318 | 3.2802 | 0.7432 | -1.7599 | 2.103 | -0.7714 | 1.8347 |
| -1.6045 | 1.7156 | 0.6686 | 2.9912 | -1.0407 | -0.5876 | 1.454 | -0.9537 | -1.1249 | 0.3589 | 2.1611 | -0.0717 | 0.7407 | 2.9084 | -0.2687 | -0.4179 | 1.2537 | -0.0727 | 3.2713 | -2.1814 | -0.0039 |
| -1.1171 | 1.6282 | 1.6519 | 1.2386 | -0.5319 | -1.272 | -1.7928 | 0.906 | -0.0132 | -0.2239 | -0.4343 | 2.121 | 0.7317 | -0.5091 | 0.658 | -0.5733 | 1.5835 | 1.1858 | 2.3282 | -0.9177 | -1.6181 |
| 1.308 | -0.0886 | 0.1474 | -1.0582 | -1.6323 | -2.0012 | 0.3547 | -1.1413 | -0.5881 | 3.3589 | -0.7384 | -1.75 | 1.5166 | -1.2541 | 0.3876 | 0.8739 | -0.0909 | -0.3714 | -1.1395 | -1.5573 | -1.0718 |
| 1.0004 | -1.2598 | -1.2799 | -2.4598 | -1.2278 | -1.1267 | 0.4904 | 3.5063 | -1.6776 | -0.6639 | 1.9531 | -1.5275 | 0.9553 | 1.827 | 0.6701 | 0.7659 | -0.2618 | -0.4465 | -1.3316 | -1.3988 | 0.0053 |
| -1.3254 | -0.3173 | -0.6868 | -1.5803 | 0.4451 | 2.5182 | -1.3199 | -0.408 | 2.1024 | -1.9498 | 1.9298 | 1.5093 | 0.9324 | 0.8315 | -0.7523 | -1.2496 | -0.2929 | 0.3467 | -0.8498 | 0.6323 | 3.4184 |
| 0.5914 | -1.5093 | -1.004 | -0.0855 | -3.3906 | 1.7148 | 1.1931 | -2.4093 | 1.3875 | 0.9472 | 0.4527 | 0.8197 | -2.0437 | 2.4092 | -1.9302 | 1.1639 | -0.6284 | -1.0526 | 1.2268 | -1.8175 | 1.3354 |
| -1.2982 | 2.4242 | 0.2467 | 3.2382 | 0.6424 | -1.4531 | 0.7048 | 1.6837 | -0.6429 | -0.9649 | -1.8163 | 0.0558 | 0.2612 | 1.9252 | -1.7063 | -1.8467 | 2.5224 | 1.2036 | 3.2031 | 0.1638 | -0.6619 |
| 1.0344 | -2.1615 | 1.7811 | -0.7519 | 0.5494 | -1.7661 | 0.2662 | 0.5671 | -1.196 | -1.0847 | 1.5234 | 2.4809 | -0.4644 | 0.5054 | 3.6858 | -0.7116 | -1.1522 | 1.5437 | -0.4629 | 1.8047 | -1.8683 |
| 2.377 | 0.1529 | 1.9666 | -0.9648 | -0.3778 | 0.0309 | -1.4341 | -1.8961 | 0.1083 | 1.4905 | 0.0819 | 0.3073 | 1.5931 | -0.595 | -1.5833 | 1.7279 | -0.2779 | 1.7889 | -1.4974 | 0.5594 | 0.4158 |
| 1.1849 | 1.7862 | -0.4284 | -1.5166 | -0.6297 | -1.3491 | 0.0954 | 0.112 | -0.3881 | -0.6619 | 3.2357 | 0.9372 | -1.0786 | 0.4355 | -1.254 | 0.7675 | 0.7941 | 0.3536 | -1.3855 | -1.4935 | -2.0233 |
| 0.6459 | 0.926 | -1.4477 | -1.1992 | -1.8761 | -1.6382 | -1.2835 | -0.6038 | 3.6995 | -1.6493 | -1.0419 | 1.188 | -2.3106 | 0.6872 | 1.389 | -0.5141 | 0.0832 | 0.3106 | -1.6527 | -1.1051 | -0.7 |
| -1.3186 | -1.0116 | 0.3243 | -0.393 | -0.5277 | 0.9797 | 2.851 | -1.665 | 0.1791 | 2.3363 | -1.6416 | 1.4658 | 1.99 | 0.0841 | 0.3065 | -0.2972 | -0.4148 | 1.2989 | -1.5903 | -0.2979 | 0.9391 |
| -0.2267 | -0.0347 | -0.7735 | 0.2873 | 1.0097 | -3.3306 | 0.0502 | 1.6933 | -1.9926 | 1.2171 | 0.8693 | 0.9492 | 0.7196 | -1.284 | -1.0306 | 0.1501 | 1.1962 | 0.0725 | 0.596 | 2.7949 | -2.2386 |
|  |  |  |  |  |  |  |  |  |  |  |  |  |  |  |  |  |  |  |  |  |
|  |  |  |  |  |  |  |  |  |  |  |  |  |  |  |  |  |  |  |  |  |
| = |  |  |  |  |  |  |  |  |  |  |  |  |  |  |  |  |  |  |  |  |
|  |  |  |  |  |  |  |  |  |  |  |  |  |  |  |  |  |  |  |  |  |
| 0.8496 | -2.1073 | 1.4218 | 2.1514 | 1.4838 | 0.9429 | -2.0179 | 2.4284 | -3.1717 | 0.7134 | -1.823 | -1.4429 | 0.4396 | -0.523 | 2.9984 | 0.5562 | -0.8016 | 0.9487 | 1.6702 | 3.0926 | 1.9329 |
| 1.8467 | 1.7962 | -0.0985 | -0.218 | -1.9617 | 0.9134 | 0.6875 | 1.4731 | -1.5344 | -0.2141 | 2.9724 | 0.4674 | 4.0818 | 0.657 | -0.3597 | 1.7213 | 1.2973 | 0.0133 | 0.2604 | -1.1869 | 1.0491 |
| -0.1198 | 0.3436 | -2.3369 | -0.7684 | 0.8799 | 3.4342 | 0.0283 | 0.244 | 1.8869 | -0.1405 | -0.2506 | 1.4864 | 1.1304 | 1.698 | -0.7652 | 0.9212 | -0.0236 | -1.4923 | 0.543 | 1.6749 | 2.9682 |
| -1.7631 | 0.1809 | 0.3429 | 1.2741 | -0.2091 | -0.0679 | 1.8058 | 0.7823 | -1.3549 | 1.7155 | -0.198 | 2.1735 | 2.1555 | 2.6712 | -1.1679 | -0.8923 | 0.9783 | -2.0867 | 1.1293 | -0.6069 | 0.2686 |
| -1.3317 | 0.0844 | -0.743 | -1.3243 | 3.3001 | 0.1874 | -1.423 | 0.2826 | -1.9532 | 1.2236 | 1.211 | 0.9995 | -1.2957 | -0.5178 | -0.8981 | -2.2615 | -0.2806 | -0.464 | -2.0593 | 2.8761 | 1.2644 |
| -0.5065 | -1.5019 | 2.7356 | -1.7085 | -0.5195 | 2.9699 | -1.0293 | 0.1613 | 2.5761 | 2.1129 | 1.4847 | -0.3248 | -1.0231 | -1.0981 | -1.4314 | 0.1817 | -2.2536 | 2.3096 | -1.3875 | 0.1218 | 2.0365 |
| 2.9005 | -1.2212 | 0.8795 | 2.2609 | -0.9453 | -0.0895 | 1.5516 | 1.569 | 1.1117 | -0.3406 | 0.646 | 0.4014 | -1.4551 | 0.288 | 1.3155 | 2.0084 | -0.5769 | -0.6968 | -0.8183 | -1.1926 | -0.1303 |
| -0.0423 | 0.1258 | -1.9145 | 0.7609 | 1.217 | 1.7493 | 0.6227 | -2.1969 | 1.2853 | -1.5138 | 0.6725 | -0.5841 | 0.4949 | 1.0368 | -1.6546 | -0.0158 | -1.5249 | -1.3783 | 1.2395 | 0.0072 | 3.3827 |
| -1.651 | 1.8012 | 2.8059 | 1.111 | 0.0067 | -2.7023 | 1.7692 | -1.6836 | 0.5492 | -2.3076 | -1.5199 | 2.9058 | -0.9238 | 2.1446 | 0.3495 | -0.5525 | 1.0879 | 1.8992 | 1.7396 | 1.4751 | -1.3513 |
| 0.0742 | 0.5628 | -0.1273 | -1.7219 | 0.3583 | 1.2529 | 2.9716 | -2.1798 | -0.3949 | 3.1781 | 0.3763 | -0.1061 | 1.8858 | -0.4551 | 1.8683 | 0.3047 | 0.3907 | 0.8341 | -1.1305 | 0.69 | 0.3456 |
| 0.7769 | -1.6269 | -0.7135 | 0.4231 | 2.5049 | 0.2554 | 0.0063 | 1.5378 | 0.0004 | -0.6642 | 1.471 | 0.1646 | 2.4395 | -0.0312 | 1.1827 | 0.0144 | -0.5684 | -0.1388 | 0.1559 | 2.191 | -0.7757 |
| -1.6423 | 0.3796 | 0.0865 | 0.2231 | -0.8028 | 2.0498 | 0.8635 | -0.7223 | 0.7953 | -0.8062 | 2.0288 | 1.08 | 2.4422 | -1.2995 | -1.883 | 0.7666 | -0.5304 | -0.7 | 0.1799 | -0.0279 | 2.5142 |
| -0.5906 | -0.6347 | -1.2086 | 2.9859 | -0.7228 | -1.4774 | 1.7083 | -1.3998 | 0.2246 | 1.1839 | 0.7409 | 0.2628 | -0.5687 | -1.1176 | -1.8292 | -0.3174 | -0.09 | -1.9126 | 2.9003 | -0.6456 | 0.0268 |
| -0.4669 | 2.8761 | -1.3698 | -0.3082 | 2.7192 | -0.3325 | -0.1191 | 3.1368 | 1.3059 | 1.1617 | -0.2146 | 1.0436 | -0.9406 | -1.7438 | 0.4857 | -0.9818 | 1.4283 | -1.8617 | -0.3928 | 1.858 | -0.3365 |
| -0.5087 | 0.3359 | 1.5381 | -0.5765 | -0.1542 | 1.6912 | 1.398 | 1.397 | -1.101 | -1.6291 | 0.0176 | 0.5057 | 0.6753 | 0.4386 | 2.4351 | -0.242 | -0.8888 | -1.0738 | -0.4752 | -0.1998 | 0.6214 |
| 0.3259 | -1.6493 | 1.0157 | 1.1477 | 1.277 | 0.9988 | -2.3599 | 2.0489 | -2.2267 | 0.9723 | -0.4061 | -2.0377 | 1.6763 | -0.814 | 1.9882 | 0.2093 | -0.895 | 0.685 | -0.1552 | 3.4361 | 1.7033 |
| 1.8126 | 2.4429 | 0.8263 | -0.0669 | -2.6572 | 1.203 | -0.1173 | 0.633 | -2.2934 | -1.9621 | 3.4359 | 0.0622 | 3.7036 | -0.068 | -0.2748 | 2.1234 | 1.4155 | 0.3784 | 0.7333 | -1.7739 | 1.5526 |
| 1.2941 | 0.2198 | -2.1187 | 0.1587 | 1.0829 | 3.0872 | -1.0124 | 0.7501 | 2.4133 | 0.0111 | -0.856 | 0.9641 | -0.2123 | 1.1517 | -0.8691 | 0.4814 | 0.2264 | -0.8594 | 0.7237 | 1.0861 | 3.6531 |
| -1.058 | 0.0118 | -0.2887 | 2.0208 | -0.5311 | -0.0334 | 1.6673 | -0.0818 | -1.3015 | 2.3678 | -0.3786 | 2.0563 | 0.598 | 2.3881 | -0.3683 | -0.738 | 0.4825 | -0.7971 | 2.3744 | -1.3933 | 0.1508 |
| 0.3987 | -0.2817 | 0.1868 | -1.2114 | 2.9916 | 0.764 | -0.5649 | 0.4754 | -1.3897 | 1.2548 | 1.0648 | 0.6505 | -0.6389 | -0.8881 | -1.0319 | -0.7414 | -0.5101 | 0.561 | -0.9726 | 2.4827 | 1.5986 |
| -0.4346 | -1.8617 | 2.5226 | -1.2488 | -0.5892 | 2.3522 | -0.6415 | 0.3047 | 1.4295 | 2.2365 | 0.4581 | 0.0176 | -1.231 | -1.4701 | -1.0651 | -0.4335 | -2.4781 | 1.9969 | -1.0658 | 0.6285 | 1.5223 |
| 2.3742 | -1.6174 | 0.7602 | 2.8616 | -0.409 | -0.7018 | 2.4729 | 0.9486 | 0.9729 | -0.1617 | 0.8516 | 0.473 | -2.2106 | 0.3092 | 0.6694 | 1.1642 | -0.966 | -0.1032 | 0.94 | -0.775 | -0.634 |
| 0.2129 | 0.4439 | -1.1999 | -0.1531 | 1.5159 | 1.6537 | 1.3294 | -1.7321 | -0.638 | -0.618 | 0.6873 | 0.0928 | 0.2081 | 1.4602 | -1.8362 | -0.1768 | -1.4448 | -0.7169 | -0.3559 | -0.1432 | 3.0261 |
|  |  |  |  |  |  |  |  |  |  |  |  |  |  |  |  |  |  |  |  |  |
|  |  |  |  |  |  |  |  |  |  |  |  |  |  |  |  |  |  |  |  |  |
| = |  |  |  |  |  |  |  |  |  |  |  |  |  |  |  |  |  |  |  |  |
|  |  |  |  |  |  |  |  |  |  |  |  |  |  |  |  |  |  |  |  |  |
| -1.6241 | 1.7155 | -1.8983 | -0.6445 | -0.4177 | 0.8514 | -0.4173 | -0.291 | 2.7552 | 1.6413 | -0.0757 | 0.8177 | 0.7905 | 2.0229 | 2.9143 | -0.7176 | 2.1674 | -1.4149 | -0.8723 | -0.6783 | 0.6477 |
| 0.103 | 0.3077 | -0.8741 | 0.7251 | 3.5005 | 1.4274 | 5.2079 | 2.002 | 0.2462 | 2.1441 | 1.3831 | -0.7944 | 1.658 | -1.0278 | 0.4358 | 0.6485 | -0.1164 | -1.5104 | 0.2821 | 3.7207 | 0.0673 |
| -1.5983 | 1.1655 | 2.0244 | 0.4475 | -0.4375 | 0.3049 | 2.2988 | 3.0189 | -0.539 | 1.1443 | -0.4192 | -0.9352 | 0.0089 | 2.7258 | 1.6962 | -0.9065 | 2.1727 | 3.393 | 0.125 | -0.1521 | 0.2729 |
| 2.5092 | 1.0436 | -0.489 | 0.0568 | -0.7761 | 0.8152 | 2.1293 | 3.4325 | -1.0549 | -0.0644 | 1.1076 | -0.0491 | 0.1449 | -0.2025 | 0.3893 | 2.2309 | 1.2704 | 0.8687 | 0.2589 | -0.0333 | 1.3845 |
| 0.0289 | 1.7745 | -1.7018 | 0.8086 | 2.3123 | 0.7397 | -1.7963 | -0.7737 | -0.1027 | -0.6164 | 0.0266 | 0.3547 | -1.7754 | 3.1498 | 1.2448 | 1.0172 | 1.7868 | -1.1289 | 0.6209 | 1.9118 | 1.6963 |
| -0.9888 | 0.7177 | 3.329 | 1.5218 | 0.9686 | 0.6652 | 0.0801 | -0.5416 | -1.1951 | 0.9485 | -1.9627 | 2.1306 | -0.6765 | 1.33 | 1.8302 | -0.8514 | 1.1661 | 2.0629 | 2.5601 | 1.8162 | 0.71 |
| 0.8646 | 1.6344 | 0.6342 | -1.4339 | 1.384 | 0.864 | -0.1593 | 1.3846 | 1.5292 | 1.5576 | -0.6088 | 0.899 | -0.8181 | -0.7492 | -0.4898 | -0.5327 | 2.2853 | 1.2973 | -1.0184 | 1.2349 | -0.1533 |
| 1.7908 | -2.0701 | 1.5617 | -1.1476 | 0.2044 | -0.9287 | 0.9237 | 0.791 | -0.031 | -0.0064 | -2.1116 | -1.3488 | 0.0794 | -0.5167 | 1.633 | 2.463 | -1.2664 | 1.3568 | -0.2785 | 0.3236 | -1.1027 |
| 1.63 | -2.1503 | -1.0687 | -1.6215 | 0.6104 | 3.0067 | -0.7551 | 4.6277 | 1.7952 | 0.3439 | 1.3054 | 2.459 | 1.4541 | 2.9041 | -1.3689 | 1.2259 | -0.5006 | -1.0927 | -0.7962 | 1.2688 | 3.1092 |
| 2.961 | -1.1597 | 1.1186 | 3.6423 | 0.6766 | -0.4639 | 2.8635 | 0.1426 | 2.1433 | 1.4412 | 0.3213 | 0.5445 | -1.3706 | 0.0562 | 1.2644 | 1.6469 | -1.7767 | 2.0056 | 4.7995 | 0.5238 | 0.4301 |
| 0.6114 | 1.6867 | 0.9145 | -0.8572 | 0.2027 | -1.4279 | 2.7311 | 0.2174 | 1.8369 | -0.6076 | -0.3999 | -0.2383 | 2.0371 | 1.5815 | -0.3208 | 1.441 | 1.7339 | -0.0264 | -0.4589 | 0.9265 | -0.4271 |
| 0.94 | 0.0167 | 0.256 | -1.1072 | 1.3554 | 1.5982 | 3.0974 | -1.4306 | -1.2399 | 0.7596 | -0.0319 | -1.0084 | -0.3074 | -0.6087 | 2.5871 | 1.2775 | 0.7579 | 1.3623 | -0.596 | 0.8188 | 0.6702 |
| 1.8927 | -1.5436 | 0.0223 | 2.1588 | 0.9356 | 0.628 | -1.156 | 0.1204 | -0.9525 | -0.7535 | 1.4666 | -1.9486 | 3.2711 | 0.8234 | 0.9208 | 2.026 | -0.864 | 0.593 | 2.0006 | 2.0612 | 1.737 |
| -0.5018 | 3.793 | 1.5385 | 1.4349 | 0.1898 | 1.8812 | -0.1814 | -1.5221 | 1.2925 | -1.0886 | 1.6493 | -0.9421 | 1.3207 | 1.6502 | -1.2413 | 0.2611 | 2.3876 | 3.0879 | 1.6224 | 0.3445 | 1.4956 |
| 2.2949 | 1.1712 | -1.7789 | 0.2674 | 0.1656 | 0.9489 | 2.0165 | 1.2622 | 0.9067 | -0.0248 | -0.3888 | -0.5355 | -1.3097 | -0.192 | -1.2124 | 1.4442 | 0.9113 | -1.7704 | -0.1559 | -0.5002 | 1.4192 |
| -1.9445 | 1.3071 | -1.3914 | -0.0383 | -0.623 | 0.321 | 0.7702 | 0.295 | 2.5861 | -0.5885 | -1.2911 | -0.1342 | -0.8087 | 2.0358 | 2.8737 | -1.1744 | 1.4218 | -0.3972 | -0.0024 | -1.159 | 0.3047 |
| -0.4752 | -0.918 | -1.4914 | 0.4866 | 3.3026 | 0.7872 | 4.4332 | 2.1169 | 0.1092 | 2.0333 | 1.9561 | 0.005 | 1.5154 | -1.0756 | 0.9742 | -0.02 | -0.7187 | -1.4075 | 1.2544 | 4.1413 | 0.5424 |
| -1.1819 | 0.1234 | 3.2587 | 0.5356 | -1.1714 | 0.8271 | 0.5651 | 2.0144 | -0.7018 | 0.8482 | 0.2101 | -1.2127 | 0.3925 | 1.7189 | 2.2668 | -1.1468 | 1.3538 | 4.4055 | -0.1744 | -0.1023 | 0.0475 |
| 1.6835 | 1.0183 | -0.4631 | 0.5988 | -1.5369 | 0.6624 | 1.3626 | 2.6113 | -0.4057 | -0.7263 | 0.7948 | 1.0515 | 1.4041 | 0.149 | 0.2667 | 2.1192 | 0.9717 | -0.1959 | 0.297 | -0.3271 | 1.4286 |
| -0.403 | 0.3125 | -2.2047 | 0.6296 | 1.4142 | 1.0066 | -1.299 | -0.4255 | -0.7923 | -0.0724 | -0.2763 | 0.5802 | -1.7406 | 2.9241 | 1.4584 | 0.7057 | 1.1759 | -0.6509 | 0.2179 | 0.7861 | 1.6902 |
| -1.3357 | -0.1971 | 2.4246 | 1.4887 | 0.8956 | 0.4094 | -0.1959 | -0.5767 | -0.9739 | 1.6725 | -2.0215 | 2.529 | -0.1316 | 0.9714 | 1.3918 | -0.7972 | -0.1546 | 2.0633 | 2.497 | 1.9997 | 0.2092 |
| 3.7462 | 1.7537 | 0.9996 | -0.4652 | 1.3798 | 0.8673 | -1.4545 | 1.4337 | 1.1225 | 2.2314 | -0.7657 | 1.0948 | 1.4133 | -0.6409 | -0.1382 | 1.6097 | 2.5373 | 0.9996 | -0.586 | 1.6238 | -0.7433 |
| 1.7947 | -1.8271 | -0.2154 | -0.4517 | 0.7375 | -0.8852 | 1.0784 | 0.26 | -0.42 | 0.0725 | -1.7425 | -0.9107 | -0.4242 | -0.9939 | 1.7699 | 1.8572 | -1.9909 | 0.2367 | -0.2937 | 0.46 | -0.9294 |
